# Optimization of cereal productivity and physiological performance under desert conditions: varying irrigation, salinity and planting density levels

**DOI:** 10.3389/fpls.2025.1488576

**Published:** 2025-03-06

**Authors:** Pedro García-Caparros, Abdullah J. Al-Dakheel, Maria D. Serret, Jose L. Araus

**Affiliations:** ^1^ Section of Plant Physiology, Faculty of Biology, University of Barcelona, Barcelona, Spain; ^2^ International Center for Biosaline Agriculture, Dubai, United Arab Emirates; ^3^ Department of Integrative Agriculture, United Arab Emirates University, Al Ain, United Arab Emirates; ^4^ AGROTECNIO (Center for Research in Agrotechnology), University of Lleida, Lleida, Spain

**Keywords:** carbon isotope composition, finger millet, *Hordeum vulgare*, nitrogen isotope composition, triticale, yield

## Abstract

Adequate irrigation with low-quality water, aligned with the specific water requirements of crops, will be critical for the future establishment of cereal crops on marginally fertile soils. This approach is essential to support global food security. To identify suitable cereal species and genotypes for these challenging conditions with the aim of optimizing yield and resilience, three different cereal species were tested under sandy soil conditions at the experimental fields of ICBA (Dubai, UAE). The experimental design employed a factorial combination split-plot arrangement including five primary factors: crop species (barley, triticale and finger millet), genotypes (3 in barley, 3 in triticale and 2 in finger millet), salinity levels (2 and 10 dS m^-1^), irrigation levels (100%, 150%, and 200% ETo), and planting densities (30 and 50 cm of spacing between rows). Agronomic parameters (e.g. plant height, grain yield, total plant dry weight and harvest index) and physiological parameters [Normalized Difference Vegetation Index (NDVI) readings, together with nitrogen and carbon concentration isotopic composition, chlorophyll, flavonoids, and anthocyanins concentrations in flag leaves and the Nitrogen Balance Index (NBI)] exhibited distinct genotypic responses across the species investigated. Regarding grain yield, salt stress did not impact barley and finger millet, whereas triticale experienced a reduction of nearly one third of its yield. Increased irrigation led to higher grain yields only in barley, while increased planting density significantly improved grain yield across all species examined demonstrating its potential as a simple agronomic intervention. Physiological responses highlighted reduced nitrogen isotope composition under both salt stress and higher planting density in all species. Nevertheless, the response to irrigation varied among species exhibiting significant negative correlations with aerial plant dry matter. In contrast, carbon isotope composition did not display a clear pattern in any of the species studied under different agronomic treatments. These results underscore the importance of selecting salt and drought tolerant species and optimizing planting density to maximize productivity on marginal soils. Future research should focus on refining irrigation strategies and identification of high-performing genotypes to improve cereal cultivation in arid regions, contributing to global food security.

## Introduction

Projections regarding climate change in forthcoming decades portend rises in global temperatures, alongside the increase in frequency and duration of drought periods across various regions. These climatic shifts, coupled with the growing need to implement supplemental irrigation as a strategy to mitigate the adverse effects of climate change in arid regions, are expected to accelerate the reliance on brackish water as an alternative irrigation source. However, this reliance is expected to impede plant growth and reduce agricultural productivity (Minhas et al., 2020; Devkota et al., 2022).

In arid and semi-arid regions, water scarcity and the progressive salinization of irrigation water represent critical constraints on plant productivity (Chamekh et al., 2016). Drought stress induces a wide range of morphological, physiological, biochemical, and molecular changes in both below-ground and above-ground tissues of cereal crops. For example, water deficit can lead to a reduced photosynthetic area and accelerated leaf senescence during the late grain-filling stage, which consequently affects crop yield (Ben Mariem et al., 2021; Toulotte et al., 2022). Accurate irrigation scheduling and precise measurement of crop water requirements are indispensable for effective water management in agriculture. The application of irrigation water based on reference evapotranspiration (ETo) is critical for the conservation of water resources. Adequate fulfillment of crop water requirements would result in enhanced growth and increased yield (Alotaibi et al., 2023; Bashir et al., 2023). Despite these measures, water scarcity in arid regions compels the use of low-quality water sources with high NaCl concentrations as the sole alternative for crop irrigation. Several studies have demonstrated the feasibility of cultivating cereal grains such as barley, triticale, and finger millet in arid and semi-arid regions using brackish water (Hammami et al., 2016; Mukami et al., 2020; Kankarla et al., 2020). Nevertheless, salt stress adversely affects the overall performance of cereal crops by promoting whole-plant senescence and reducing the remobilization and transfer of pre-stored assimilates from vegetative tissues to grains. This results in a decreased grain filling rate, reduced grain weight, and consequently a marked decline in yield (Shahbaz and Ashraf, 2013; Kumar et al., 2022). The levels of salt tolerance vary among cereal species and even among cultivars. Previous studies indicate that barley demonstrates relatively high salinity tolerance, while finger millet and triticale exhibit moderate tolerance to saline conditions (Zeeshan et al., 2020; Mbinda and Mukami, 2021; Mohammadi Alagoz et al., 2023).

Planting density is a critical agronomic practice that significantly influences grain yield and a range of other key agronomic traits in crops (Postma et al., 2021; Cao et al., 2022). Previous research has indicated that the optimal planting density is crop-specific varying according to the species and environmental conditions (Wilke et al., 2021; Niharika et al., 2021; Mohamed, 2023). Furthermore, different cultivars exhibit distinct responses to planting density in terms of productivity and resource use efficiency (Ghazvineh et al., 2020; Zheng et al., 2021). Enhancing planting density and optimizing light utilization represent pivotal strategies for achieving higher crop yields (Zhang et al., 2021; Ming et al., 2017). Nonetheless, augmenting planting density concurrently intensifies intra-specific competition for essential resources such as light, nutrients, and, particularly in arid and semiarid regions, water (MacLaren et al., 2023).

Phenotype can be defined as the set of observable characteristics of an organism, which arise from the complex interplay among its genotype, the environment, and crop management practices (Yang et al., 2020; Großkinsky et al., 2023). The relative contributions of genotype by environment and management interactions contribute to the phenotypic complexity of traits such as yield (Araus et al., 2018, 2023). In modern agriculture, the non-invasive assessment of plant traits such as crop growth, potential photosynthetic capacity, and water status is gaining increasing importance for optimizing crop performance and resource use efficiency (Yang et al., 2017; Vargas et al., 2020).

Incorporating remote sensing methodologies alongside targeted laboratory techniques, such as the analysis of stable isotope signatures, presents a promising avenue for enhancing the predictive efficacy of phenotyping processes under arid and semi-arid conditions (Gracia-Romero et al., 2019; Rezzouk et al., 2020a). Stable carbon and nitrogen isotope compositions in plants offer valuable, time-integrated indicators of their physiological responses and interactions with abiotic and biotic environmental factors (Dong et al., 2022; Rezzouk et al., 2022). Evidence from various studies indicates that abiotic stresses, such as salinity and drought, can result in either an increase or a decrease in the carbon and nitrogen isotope composition of cereal grains (Lopes et al., 2004; Lopes and Araus, 2006; Yousfi et al., 2009, 2012). This isotopic response varies on the plant`s photosynthetic pathway, differing between C_3_ crops, such as barley and triticale, and C_4_ crops, such as finger millet (Aranibar et al., 2008; Murphy and Bowman, 2009). The carbon isotope composition (δ^13^C) serves as a temporally integrated indicator for elucidating the interplay between intracellular and atmospheric carbon dioxide (CO_2_) ratios (Ci/Ca) attained by the plant throughout the photosynthetic process. The Ci/Ca ratio reflects the equilibrium between stomatal conductance and the photosynthetic activity of the plant (Wallace et al., 2013; Vernooij et al., 2021). The total carbon content in crops represents the assimilation of atmospheric CO_2_ by plant chloroplasts during the pivotal process of carbon fixation. Similarly, the nitrogen isotope composition (δ^15^N), in conjunction with the total nitrogen (N) content in plant biomass, serves as a dual indicator elucidating the influence of growing conditions on the intricate nitrogen metabolism of the plant organism (Vicente et al., 2019; Rezzouk et al., 2020b). Nevertheless, the precise mechanisms and functional roles underlying these processes remain incompletely understood (Pritchard and Guy, 2005; Coque et al., 2006).

Cereals constitute a significant proportion of global plant-derived food production and represent a predominant category among harvested crops. Barley is generally used as both food and fodder, whereas triticale and finger millet are widely used as animal feed (Luo et al., 2019; Pour-Aboughadareh et al., 2021; Hussain et al., 2023). In arid and desert regions, where fodder scarcity represents significant challenges, these crops may represent a strategic alternative. Nevertheless, the ongoing impacts of climate change have heightened the influence of abiotic stressors, consequently exacerbating yield attenuation and potentially compromising global food security. Within this paradigm, improvement of agricultural practices and enhancing crop adaptability to water scarcity and rising salinity levels in soils and irrigation water assume pivotal significance (Wang et al., 2018). A comprehensive review of the existing literature reveals a plethora of studies focused on investigating the individual impacts of water deficit, salinity, planting density, and varietal differences on yield performance and physiological responses in barley, finger millet, and triticale cultivation (Soleymani and Shahrajabian, 2011; Hasanuzzaman et al., 2019; Zeeshan et al., 2020; Mukami et al., 2019, 2020; Amulya Manasa and Umesha, 2022; Mohammadi Alagoz et al., 2023; He et al., 2024). Nevertheless, there is a notable gap in the literature regarding the combined effects of these agricultural practices and abiotic stressors on these crops, especially in arid regions. The usual situation under field conditions is the appearance of simultaneous stress, rather than the effect of a single stress. Our research hypothesis proposes that the combined influence of these factors may result in a greater impact on crop productivity and physiological performance compared to the direct effect of each factor independently. Consequently, the primary objective of this study was to ascertain the maximum attainable biomass and grain productivity as well as to discern the key physiological attributes, across various cultivars of barley, finger millet, and triticale. This was achieved by evaluating their performance under diverse management conditions, which incorporated the interactions between different water salinities, irrigation levels, planting densities and multiple genotypes per species.

## Materials and methods

### Plant material and growth conditions

The experiment was conducted at the field facilities of the International Center for Biosaline Agriculture (ICBA) in Dubai, United Arab Emirates (25°05′49′′ N, 55°23′25′′E). Meteorological data, comprising temperature values and precipitation measurements, were systematically collected from the Dubai International Airport meteorological station (https://meteostat.net/en/station/41194?t=2019-08-01/2019-10-31) and are showed in **Supplementary Figure S1**. During the experimental period, mean daily temperature values ranged from 15.6°C to 29.7°C, while the mean daily rainfall ranged from 0 to 11.7 mm. The soil characteristics at ICBA’s experimental fields were predominantly sandy, with a fine sand composition of 98%, minimal silt (1%), and clay content (1%). The soils were calcareous, with calcium carbonate equivalents ranging from 50 to 60%, highly porous (45% porosity), and moderately alkaline, with a pH of 8.22. Organic matter content was notably low, measuring less than 0.5%. The soil exhibited a saturation percentage of 26, indicative of a high drainage capacity, and an electrical conductivity of the saturated extract (ECe) of 1.2 dS m^-1^. According to the American Soil Taxonomy system (Soil Survey Staff, 2010), the soil type was classified as Typic Torripsamments, characterized by its carbonate-rich nature and hyperthermic conditions (Shahid et al., 2009). To meet crop nutrient requirements, organic fertilizer (N 1.5%, K 1.65%, Na 1.22%, pH 7.7, C/N ratio 16.5, organic matter 41% and moisture content 1.64%) was applied at a rate of 30 t ha^-1^, incorporated into the soil. Sowing was performed manually on November 29 and 30, 2016. During the subsequent two weeks, drip irrigation using fresh water with an electrical conductivity of 1 dS m^-1^, was applied to ensure unimpeded germination processes, as recommended by Koyro and Eisa (2008). Following this establishment phase, the salt and irrigation treatments were initiated and maintained throughout the crop cultivation cycle.

### Experimental design and treatments

The experimental design employed a factorial combination split-plot arrangement with three replications per treatment, including five primary factors: crop species, genotypes, salinity levels, irrigation levels, and planting densities. Salinity and irrigation manipulations were administered via the Supervisory Control and Data Acquisition (SCADA) system. Salinity levels were categorized into two conditions: S1 denoting low electrical conductivity (EC) levels ranging from 1-2 dS m^-1^, and S2 corresponding to high EC levels ranging from 8-10 dS m^-1^. Irrigation levels were divided into three categories: I1 supplying 100% of the reference evapotranspiration (ET_0_), I2 supplying 150% ET_0_, and I3 supplying 200% ET_0_. Planting densities were specified as D1, with 30 cm row spacing, and D2 with 50 cm row spacing. Within the barley genotype category, three accessions were evaluated: C1 (N2-35), C2 (N2-4), and C3 (IPA7). Similarly, three triticale accessions were examined: C1 (PI388678), C2 (PI4295152), and C3, a variety originating from Jordan and sourced from Syria. Additionally, two finger millet genotypes were included in the study: C1 and C2, both representing local cultivars from Yemen. The selection of these genotypes was based on the germplasm resources accessible in ICBA and cultivated in the region.

The plot dimensions were set at 2 × 2 m, with a plant-to-plant distance of 25 cm and inter-row spacing set at either 30 or 50 cm, depending on the planting density treatments. Drip irrigation was employed throughout the experimental period, with drippers positioned at 25 cm intervals. The discharge rate from each dripper was calibrated to 4 L h^-1^ per plant. The irrigation period varied based on climatic conditions and the crop development stage, ranging from the full tillering to the dough-making stage. Manual weeding practices were implemented throughout the entire crop cycle as required, without the use of herbicidal agents. To mitigate grain losses during the grain filling period, a net with a mesh size of approximately 15 × 15 mm^2^ was installed to prevent the entry of small birds.

### Biomass and yield parameters

Yield and biomass parameters were evaluated using five randomly selected plants harvested from the central row of each plot upon reaching grain physiological maturity (third week of March). Plant height was recorded using a ruler, while the number of stems and spikes were counted directly. After threshing the plants, grain yield was evaluated. The determination of dry weight for stems, spikes and total plant biomass, entailed an initial sun-drying period of two days, followed by a subsequent drying in a forced-air oven at 80°C for 48 h. The harvest index (HI) was calculated as the ratio of grain yield to total plant dry weight.

### Physiological determinations

Physiological measurements and sampling for the three crops species were conducted during the period between heading and early anthesis.

### NDVI determinations

Canopy reflectance measurements were conducted utilizing a GreenSeeker hand-held Optical Sensor (Ntech Industries, Inc., Ukiah, CA, USA). This sensor is specifically designed to determine the Normalized Difference Vegetation Index (NDVI) by employing its proprietary light source. The NDVI calculation is based on the spectral reflectance measurements obtained at red (660 nm) and near-infrared (770 nm) wavelengths following the equation reported by Tucker and Sellers (1986). The NDVI is computed using the following Equation 1:


(1) NDVI=[(NIR-VIS)/(NIR+VIS)]

where NIR and VIS denote the near infrared and visible red wavelengths, respectively.

The GreenSeeker device recorded a range of 10-15 NDVI counts per plot. These counts were subsequently averaged within each plot, resulting in a singular value representative of the vegetation therein. Measurement acquisition occurred near solar noon, between 11 a.m. and 1 p.m. Data collection occurred on January 19 and February 1, 2017 (referred to as NDV1 and NDVI2, respectively) to evaluate the potential differences between two different crop phenological stages of the experimental study.

### Leaf pigment and NBI determinations

Leaf pigment concentrations were assessed in the flag leaf of the three species using a portable leaf-clip sensor (Dualex, Dualex Force-A, Orsay, France). The Dualex sensor enables non-destructive determinations of chlorophyll (Chl, in µg cm^-^²), flavonoid (Fla, dimensionless index), and anthocyanin (Anth, dimensionless index) concentration, leveraging chlorophyll fluorescence excitation spectra (Cerovic et al., 2012). Additionally, this sensor computes the nitrogen balance index (NBI), representing the Chl/Flav ratio in relation to nitrogen and carbon allocation dynamics (Cerovic et al., 2015). Following the experimental protocol, ten recently fully expanded (i.e., non-senescent) leaves were chosen from the central rows of each plot. Measurements were conducted on the adaxial surface of the leaves, with data acquisition taking place on January 19, 2017.

### Total nitrogen and carbon and stable isotope analyses

The leaves used for pigment content quantification underwent successive washing cycles with both tap and distilled water. They were then desiccated in an oven set at 60°C for a duration of two days. Once dried, the leaves were finely ground into an uniform powder. A subsample of the dried leaf powder was used to determine total carbon and nitrogen concentrations, as well as the stable isotopic signatures of carbon (^13^C/^12^C ratio) and nitrogen (^15^N/^14^N ratio). These analyses were conducted at the Scientific Facilities of the University of Barcelona. Approximately 1 mg of subsample was weighed into tin capsules and the analyses were carried out through an elemental analyzer (Flash 1112 EA; ThermoFinnigan, Schwerte, Germany) integrated with an isotope ratio mass spectrometer (Delta C IRMS, ThermoFinnigan), which operated in continuous flow mode. The ^13^C/^12^C ratios (R) of leaf material were represented in δ notation (Coplen, 2008), denoted in per mil (‰), with the sample denoting the leaf plant material and the standard representing Pee Dee Belemnite (PDB) calcium carbonate. International isotope secondary standards with established ^13^C/^12^C ratios (IAEA CH7 3, polyethylene foil; IAEA CH6, sucrose; USGS 40, l-glutamic acid) were employed, ensuring an analytical precision of 0.1‰. The identical δ notation convention was applied for expressing the ^15^N/^14^N ratio, with the standard referencing N_2_ in air (Coplen, 2008). For nitrogen, international isotope secondary standards (IAEA N1, IAEAN2, IAEANO3, and USGS40) were utilized, maintaining a precision of 0.3‰. The nitrogen and carbon content in leaves were expressed as percentages (%).

The carbon and nitrogen isotopic compositions, denoted as δ^13^C and δ^15^N respectively, were expressed utilizing the following notation according to Coplen (2008) (Equation 2).


(2) $$\delta^{13}\text{C or }\delta^{15}\text{N }\left(‰\right)=\left[\left(\text{Rsample}/\text{R standard}\right)-1\right]\times 1000$$

where δ^13^C and δ^15^N represents the ratios of isotopes ^13^C/^12^C and ^15^N/^14^N in the sample, respectively, both expressed in ‰. Meanwhile, R standard denotes the molar abundance ratio of the secondary standard calibrated against the primary standard.

### Statistical analysis

Analysis of variance (ANOVA) was conducted using the statistical software Statgraphics Centurion XVI (Statpoint Technologies, Inc. Warrenton, VA, USA) to scrutinize the impacts of the following factors across diverse species: genotypes, irrigation treatments, salt treatments, planting density, and their respective interactions. Means were compared based on the Least Significant Difference (LSD) test at the 5% probability level. Additionally, a bivariate correlation analysis was carried out using the same software to compute Pearson correlation coefficients among the analytic traits.

## Results

### Effects of salinity, irrigation, and planting density levels on agronomic components, and physiological parameters in barley cultivars

In evaluating agronomic parameters in relation to genotype variability, there were clear differences among the examined barley cultivars. Notably, the barley cultivar C1 (N2-35) showed superior performance, recording the highest values for spike dry weight and total plant dry weight. In contrast, barley C2 (N2-4) exhibited the highest harvest index among the genotypes assessed. Under conditions of increasing saline concentrations, no significant changes in the agronomic parameters of barley plants were detected. Nevertheless, an analysis of irrigation rates revealed that the supply of the lowest irrigation rate, (I100), resulted in reduced both grain yield and harvest index. Furthermore, the evaluation of planting density indicated that higher planting densities were generally associated with improvements across agronomic parameters, except for the harvest index, which showed a notable reduction (**Table 1**).

**Table 1:** Effects of cultivar (C1:; C2:; C3): salinity levels [S1: low electrical conductivity (EC) (1-2 dS m^-1^), S2: high EC (8-10 dS m^-1^)], irrigation levels (I1: 100% ET_0_, I2: 150% ET_0_ and I3: 200% ET_0_), and planting density (D1: 30 cm and D2: 50 cm of space between rows) on biomass and yield parameters in barley plants over the experimental period.

Factors			Plant height (cm)	Stem number per m^2^	Spike number per m^2^	Stem DW per m^2^	Spike DW per m^2^	Grain yield (kg ha^-1^)	TDW (kg ha^-1^)	HI (-)
**A**	**Cultivar**	**C1**	72.25 ± 7.38 ^b^	330.02 ± 86.23 ^a^	260.61 ± 78.32 ^a^	298.72 ± 150.92 ^a^	676.86 ± 261.89 ^a^	1231.67 ± 674.03 ^a^	7085.78 ± 3512.79 ^a^	0.20 ± 0.10 ^b^
		**C2**	71.22 ± 9.54 ^b^	352.56 ± 101.39 ^a^	266.94 ± 81.01 ^a^	306.19 ± 132.21 ^a^	561.42 ± 172.25 ^b^	1374.44 ± 509.09 ^a^	6204.90 ± 2658.30 ^b^	0.24 ± 0.10 ^a^
		**C3**	77.74 ± 8.63 ^a^	330.41 ± 89.27 ^a^	257.78 ± 63.05 ^a^	281.17 ± 134.47 ^a^	633.90 ± 178.39 ^ab^	1000.56 ± 513.33 ^b^	6472.99 ± 2582.27 ^ab^	0.16 ± 0.08 ^b^
**B**	**Salinity**	**S1**	73.74 ± 8.81 ^a^	332.76 ± 98.78 ^a^	253.81 ± 70.65 ^a^	256.71 ± 87.24 ^a^	597.89 ± 109.19 ^a^	1183.33 ± 712.96 ^a^	6121.20 ± 2500.77 ^a^	0.22 ± 0.11 ^a^
		**S2**	73.73 ± 9.18 ^a^	342.58 ± 85.91 ^a^	276.94 ± 68.45 ^a^	305.33 ± 63.13 ^a^	641.23 ± 136.62 ^a^	1221.11 ± 429.54 ^a^	6554.58 ± 3116.32 ^a^	0.19 ± 0.08 ^a^
**C**	**Irrigation**	**I100**	71.81 ± 5.75 ^a^	327.61 ± 86.76 ^a^	250.77 ± 71.04 ^a^	257.33 ± 138.64 ^b^	641.01 ± 264.28 ^a^	933.89 ± 354.74 ^b^	6896.11 ± 2877.45 ^a^	0.15 ± 0.06 ^b^
		**I150**	74.82 ± 9.61 ^a^	342.37 ± 86.47 ^a^	270.64 ± 72.05 ^a^	341.17 ± 136.15 ^a^	620.29 ± 168.46 ^a^	1341.67 ± 492.11 ^a^	6686.04 ± 3251.26 ^a^	0.22 ± 0.09 ^a^
		**I200**	74.58 ± 10.68 ^a^	343.02 ± 104.24 ^a^	263.92 ± 79.18 ^a^	287.58 ± 131.20 ^b^	610.88 ± 195.90 ^a^	1331.11 ± 755.68 ^a^	6181.53 ± 2714.03 ^a^	0.23 ± 0.12 ^a^
**D**	**Planting density**	**D30**	71.07 ± 8.19 ^b^	274.48 ± 61.26 ^b^	214.94 ± 46.23 ^b^	250.52 ± 99.84 ^b^	567.74 ± 192.64 ^b^	939.26 ± 348.43 ^b^	4670.93 ± 1699.96 ^b^	0.22 ± 0.10 ^a^
		**D50**	76.39 ± 8.96 ^a^	400.86 ± 72.80 ^a^	308.61 ± 66.58 ^a^	340.20 ± 157.11 ^a^	680.38 ± 216.68 ^a^	1465.19 ± 656.68 ^a^	8504.86 ± 2667.93 ^a^	0.18 ± 0.10 ^b^
Interaction										
**AB**			ns	ns	ns	ns	ns	ns	ns	ns
**AC**			ns	ns	ns	ns	ns	ns	ns	ns
**AD**			ns	ns	ns	ns	ns	ns	ns	ns
**BC**			ns	ns	ns	*	*	*	ns	ns
**BD**			ns	ns	ns	*	ns	ns	*	*
**CD**			ns	ns	ns	ns	*	*	*	*
**ABC**			ns	ns	ns	ns	ns	ns	ns	ns
**ABD**			ns	ns	ns	ns	ns	ns	ns	ns
**ACD**			ns	ns	ns	ns	ns	ns	ns	ns
**BCD**			ns	ns	ns	ns	*	*	*	ns
**ABCD**			ns	ns	ns	ns	ns	ns	ns	ns

Treatment values are the means ± standard deviation of 3 replicates per treatment. Values with different letters within a column are significantly different at p< 0.05 (analysis of variance and least significant difference test). ns: indicates non-statistical differences. * indicates statistical differences. DW is dry weight, TDW is total dry weight and HI is harvest index.

In evaluating physiological parameters across different barley genotypes, there were no clear differences, except for barley cv. C3 (IPA-7), which exhibited the highest NDVI1 readings. Under increasing salinity levels, discernible trends in physiological parameters were noted. Specifically, NDVI (1 and 2) readings and δ^15^N exhibited a decreasing trend. Conversely, salinity was positively correlated with increased N, δ^13^C, chlorophyll (Chl) and anthocyanin (Anth) concentration, and the nitrogen balance index (NBI). The impact of irrigation rates reported that the lowest irrigation rate (I100) resulted in increased N, δ^13^C, Chl concentration, and NBI. In contrast, the highest irrigation rate (I200) was associated with elevated NDVI (1 and 2) readings but resulted in a reduction in C concentration. An exploration of planting density revealed multifaceted effects on physiological parameters. Specifically, higher planting densities increased NDVI (1 and 2) readings, N and Chl concentration, and NBI. Nevertheless, δ^15^N showed an opposite trend, with its levels decreasing as planting density increased (**Table 2**).

**Table 2:** Effects of cultivar (C1:; C2:; C3): salinity levels [S1: low electrical conductivity (EC) (1-2 dS m^-1^), S2: high EC (8-10 dS m^-1^)], irrigation levels (I1: 100% ET_0_, I2: 150% ET_0_ and I3: 200% ET_0_), and planting density (D1: 30 cm and D2: 50 cm of space between rows) on physiological parameters in barley plants over the experimental period.

Factors			NDVI1 (-)	NDVI2 (-)	N concentration (%)	δ^15^N (‰)	C concentration (%)	δ^13^C (‰)	Chl (µg cm^-^²)	Flav (-)	Anth (-)	NBI (-)
**A**	**Cultivar**	**C1**	0.26 ± 0.06 ^b^	0.34 ± 0.06 ^a^	4.42 ± 0.81 ^a^	2.87 ± 3.51 ^a^	42.62 ± 1.34 ^a^	-29.21 ± 0.75 ^a^	35.24 ± 5.35 ^a^	1.32 ± 0.09 ^a^	0.10 ± 0.02 ^a^	27.43 ± 4.82 ^a^
		**C2**	0.27 ± 0.06 ^ab^	0.33 ± 0.07 ^a^	4.47 ± 0.64 ^a^	2.76 ± 3.15 ^a^	42.60 ± 0.99 ^a^	-29.24 ± 0.91 ^a^	36.07 ± 5.88 ^a^	1.33 ± 0.11 ^a^	0.10 ± 0.02 ^a^	27.67 ± 4.62 ^a^
		**C3**	0.29 ± 0.07 ^a^	0.34 ± 0.07 ^a^	4.20 ± 0.63 ^a^	3.05 ± 3.42 ^a^	42.59 ± 0.91 ^a^	-28.91 ± 0.82 ^a^	34.95 ± 7.13 ^a^	1.27 ± 0.20 a	0.10 ± 0.03 ^a^	28.44 ± 5.48 ^a^
**B**	**Salinity**	**S1**	0.29 ± 0.06 ^a^	0.37 ± 0.06 ^a^	4.26 ± 0.71 ^b^	3.43 ± 3.89 ^a^	42.54 ± 1.16 ^a^	-29.35 ± 0.89 ^b^	34.58 ± 6.03 ^b^	1.31 ± 0.11 ^a^	0.09 ± 0.02 ^b^	27.02 ± 5.26 ^b^
		**S2**	0.26 ± 0.07 ^b^	0.31 ± 0.06 ^b^	4.46 ± 0.69 ^a^	2.36 ± 2.60 ^b^	42.67 ± 1.02 ^a^	-28.88 ± 0.70 ^a^	36.30 ± 6.15 ^a^	1.30 ± 0.17 ^a^	0.10 ± 0.02 ^a^	28.73 ± 4.50 ^a^
**C**	**Irrigation**	**I100**	0.26 ± 0.06 ^b^	0.34 ± 0.06 ^ab^	4.68 ± 0.57 ^a^	1.08 ± 3.33 ^c^	43.18 ± 0.86 ^a^	-28.86 ± 0.65 ^a^	37.69 ± 6.21 ^a^	1.28 ± 0.20 ^b^	0.10 ± 0.02 ^a^	30.35 ± 3.46 ^a^
		**I150**	0.26 ± 0.06 ^b^	0.32 ± 0.06 ^b^	4.40 ± 0.51 ^b^	2.47 ± 1.90 ^b^	42.97 ± 0.99 ^a^	-29.18 ± 0.86 ^ab^	34.69 ± 5.14 ^b^	1.33 ± 0.11 ^a^	0.09 ± 0.02 ^b^	26.58 ± 4.28 ^b^
		**I200**	0.30 ± 0.07 ^a^	0.36 ± 0.07 ^a^	3.99 ± 0.81 ^c^	5.13 ± 3.26 ^a^	41.67 ± 0.72 ^b^	-29.32 ± 0.93 ^b^	34.07 ± 6.52 ^b^	1.31 ± 0.11 ^ab^	0.11 ± 0.02 ^a^	26.82 ± 5.93 ^b^
**D**	**Planting density**	**D30**	0.25 ± 0.06 ^b^	0.32 ± 0.07 ^b^	4.25 ± 0.70 ^b^	4.52 ± 2.80 ^a^	42.49 ± 1.02 ^a^	-29.08 ± 0.87 ^a^	34.60 ± 6.34 ^b^	1.31 ± 0.15 ^a^	0.10 ± 0.02 ^a^	27.17 ± 5.06 ^b^
		**D50**	0.30 ± 0.06 ^a^	0.35 ± 0.06 ^a^	4.47 ± 0.69 ^a^	1.27 ± 3.05 ^b^	42.71 ± 1.16 ^a^	-29.15 ± 0.81 ^a^	36.28 ± 5.82 ^a^	1.31 ± 0.14 ^a^	0.10 ± 0.02 ^a^	28.57 ± 4.80 ^a^
Interaction												
**AB**			*	ns	ns	ns	ns	ns	ns	*	*	ns
**AC**			ns	ns	ns	ns	ns	ns	*	*	*	ns
**AD**			ns	ns	ns	ns	ns	ns	ns	ns	ns	ns
**BC**			*	*	ns	ns	*	*	*	*	*	*
**BD**			*	*	*	*	*	*	*	ns	*	ns
**CD**			ns	ns	ns	*	*	ns	ns	ns	*	ns
**ABC**			ns	ns	ns	ns	ns	ns	*	*	*	ns
**ABD**			ns	ns	ns	ns	*	ns	ns	ns	ns	ns
**ACD**			ns	ns	ns	ns	ns	ns	ns	ns	ns	ns
**BCD**			*	*	*	ns	ns	*	*	ns	*	*
**ABCD**			ns	ns	ns	ns	ns	ns	ns	ns	ns	*

Treatment values are the means ± standard deviation of 3 replicates per treatment. Values with different letters within a column are significantly different at p< 0.05 (analysis of variance and least significant difference test). ns: indicates non-statistical differences. * indicates statistical differences.

**Figure 1:**
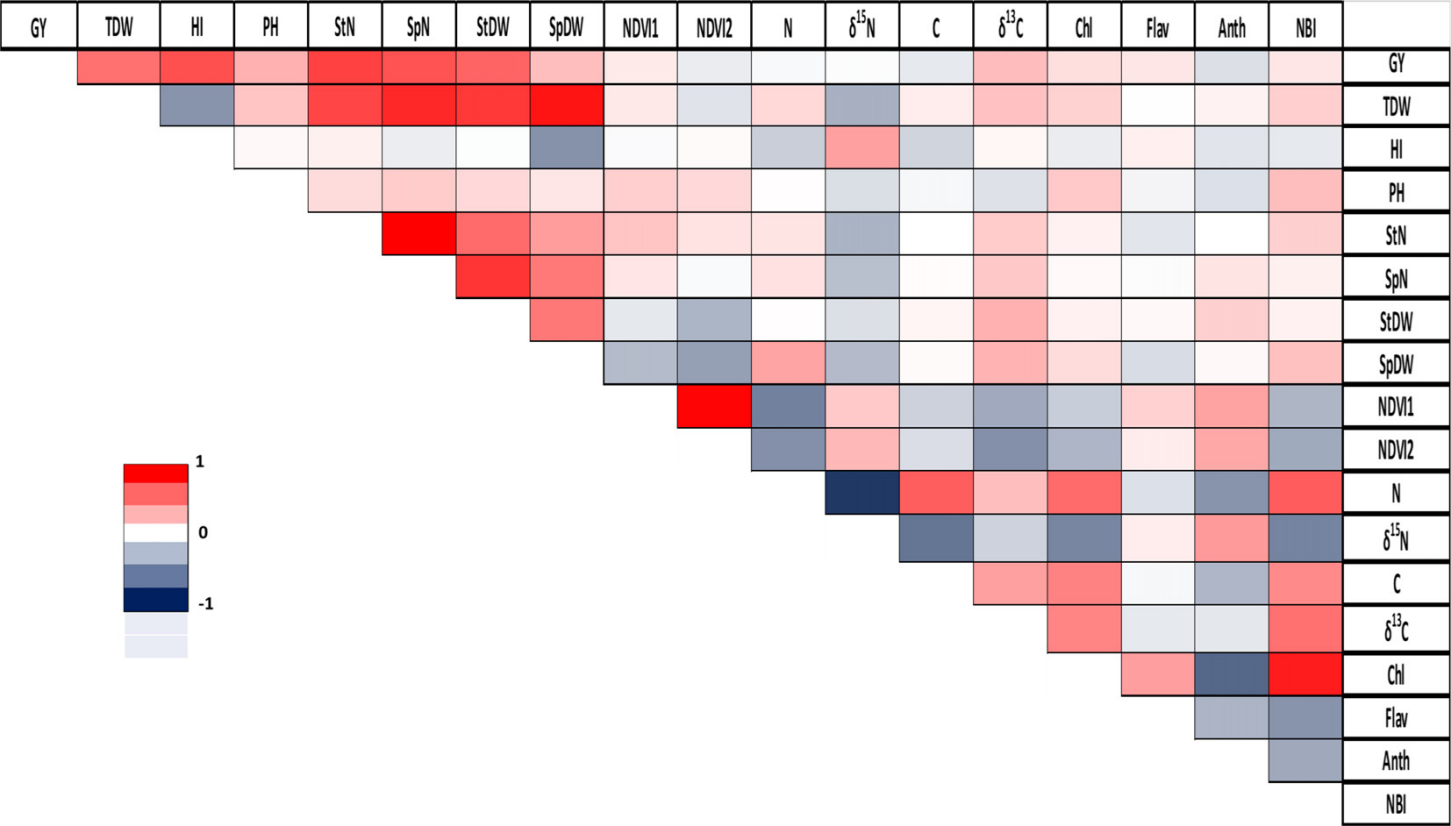
Correlation between agronomic and physiological parameters in barley. GY, grain yield; TDW, total plant dry weight; HI, harvest index; PH, plant height; StN, stem number; SpN, spike number; StDW, stem dry weight; SpDW, spike dry weight; NDVI1, greenseeker reading 1; NDVI2, greenseeker reading 2; N, total nitrogen; δ^15^N, stable nitrogen isotope composition; C, total carbon; δ^13^C, stable carbon isotope composition; Chl, Chlorophyll; Flav, flavonoid; Anth, anthocyanin; NBI, Nitrogen Balance Index. Pearson correlation coefficient (r) is represented using a color gradient, where deep red corresponds to r=1, white indicates r=0, and deep blue represents r=-1.

The analysis of correlations across all barley genotypes revealed significant positive correlations in stem number/spike number (r = 0.81), NDVI reading 1/NDVI reading 2 (r = 0.80), and total plant dry weight/spike dry weight (r = 0.75). Conversely, the strongest negative correlation was observed between N concentration and δ^15^N with a correlation coefficient of r = -0.76 (**Figure 1**). A more detailed analysis for each genotype is provided in the **Supplementary Material** (**Supplementary Figures S2–S4**).

### Effects of salinity, irrigation, and planting density levels on agronomic components, and physiological parameters in triticale cultivars

In the evaluation of agronomic parameters within the context of genotype variability, significant findings were observed, particularly in triticale cv. C2 (PI429152), which showed the lowest grain yield and harvest index among the genotypes studied. Under increasing saline concentrations, a discernible pattern emerged where only stem dry weight increased, while other agronomic parameters declined. The analysis of irrigation rates revealed different patterns in the agronomic performance of triticale plants. Notably, the lowest irrigation rate (I100) was associated with reductions in spike dry weight and harvest index. In contrast, the medium irrigation rate (I150) resulted in heightened spike number, stem dry weight, total plant dry weight, and grain yield. Furthermore, an exploration of planting density elucidated notable trends in agronomic parameters. Higher planting densities were linked to increases in all parameters studied. However, this increase in performance was accompanied by a decline in harvest index (**Table 3**).

**Table 3:** Effects of cultivar (C1:; C2:; C3): salinity levels [S1: low electrical conductivity (EC) (1-2 dS m^-1^), S2: high EC (8-10 dS m^-1^)], irrigation levels (I1: 100% ET_0_, I2: 150% ET_0_ and I3: 200% ET_0_), and planting density (D1: 30 cm and D2: 50 cm of space between rows) on biomass and yield parameters in triticale plants over the experimental period.

Factors			Plant height (cm)	Stem number per m^2^	Spike number per m^2^	Stem DW per m^2^	Spike DW per m^2^	Grain yield (kg ha^-1^)	TDW (kg ha^-1^)	HI (-)
**A**	**Cultivar**	**C1**	102.72 ± 11.82 ^a^	352.47 ± 120.64 ^a^	314.55 ± 101.65 ^a^	398.67 ± 134.54 ^a^	507.69 ± 241.36 ^a^	1854.72 ± 818.25 ^a^	6624.86 ± 3427.82 ^a^	0.31 ± 0.10 ^a^
		**C2**	101.01 ± 10.06 ^a^	368.86 ± 111.72 ^a^	329.78 ± 102.53 ^a^	387.00 ± 141.97 ^a^	549.44 ± 243.69 ^a^	1595.00 ± 807.09 ^b^	6799.07 ± 3269.79 ^a^	0.26 ± 0.10 ^b^
		**C3**	98.65 ± 9.59 ^a^	332.71 ± 109.15 ^a^	295.71 ± 95.81 ^a^	386.50 ± 143.99 ^a^	480.14 ± 157.04 ^a^	1941.11 ± 862.70 ^a^	6341.99 ± 2950.63 ^a^	0.34 ± 0.10 ^a^
**B**	**Salinity**	**S1**	104.07 ± 10.20 ^a^	380.91 ± 131.41 ^a^	330.96 ± 117.54 ^a^	336.30 ± 154.11 ^b^	570.13 ± 222.59 ^a^	2088.52 ± 1004.81 ^a^	6958.11 ± 3476.41 ^a^	0.33 ± 0.10 ^a^
		**S2**	97.52 ± 9.99 ^b^	321.79 ± 84.36 ^b^	295.73 ± 75.99 ^b^	445.15 ± 96.10 ^a^	454.72 ± 198.22 ^b^	1505.37 ± 474.35 ^b^	6219.17 ± 2877.14 ^b^	0.27 ± 0.10 b
**C**	**Irrigation**	**I100**	98.18 ± 9.09 ^a^	343.78 ± 109.55 ^a^	305.81 ± 100.97 ^b^	332.22 ± 117.60 ^c^	450.97 ± 157.06 ^b^	1449.17 ± 361.52 ^c^	6156.06 ± 2181.05 ^b^	0.25 ± 0.06 ^b^
		**I150**	102.44 ± 12.21 ^a^	372.34 ± 118.63 ^a^	339.10 ± 104.03 ^a^	461.49 ± 141.03 ^a^	547.36 ± 239.88 ^a^	2143.61 ± 1050.46 ^a^	7122.36 ± 3836.71 ^a^	0.33 ± 0.11 ^a^
		**I200**	101.76 ± 9.96 ^a^	337.92 ± 113.36 ^a^	295.14 ± 92.34 ^b^	378.44 ± 128.61 ^b^	538.94 ± 238.18 ^a^	1798.06 ± 806.08 ^b^	6487.50 ± 3359.13 ^ab^	0.31 ± 0.11 ^a^
**D**	**Planting density**	**D30**	97.76 ± 8.92 ^b^	268.33 ± 56.71 ^b^	242.11 ± 53.10 ^b^	310.04 ± 90.23 ^b^	420.07 ± 142.28 ^b^	1349.81 ± 375.99 ^b^	4209.07 ± 1542.97 ^b^	0.34 ± 0.11 ^a^
		**D50**	103.83 ± 11.27 ^a^	434.36 ± 94.38 ^a^	384.58 ± 84.04 ^a^	471.41 ± 132.72 ^a^	604.78 ± 240.84 ^a^	2244.07 ± 926.89 ^a^	8968.21 ± 2592.75 ^a^	0.25 ± 0.07 ^b^
Interaction										
**AB**			ns	ns	ns	ns	ns	ns	ns	ns
**AC**			ns	ns	ns	ns	ns	ns	ns	ns
**AD**			ns	ns	ns	ns	ns	ns	ns	ns
**BC**			ns	ns	ns	*	*	*	ns	*
**BD**			ns	*	*	ns	*	*	ns	ns
**CD**			ns	ns	ns	*	*	*	*	*
**ABC**			ns	ns	ns	ns	ns	ns	ns	ns
**ABD**			ns	ns	ns	ns	ns	ns	ns	ns
**ACD**			*	ns	ns	ns	ns	ns	ns	ns
**BCD**			ns	ns	ns	*	ns	*	*	*
**ABCD**			ns	ns	ns	ns	ns	ns	ns	ns

Treatment values are the means ± standard deviation of 3 replicates per treatment. Values with different letters within a column are significantly different at p< 0.05 (analysis of variance and least significant difference test). ns, indicates non-statistical differences. * indicates statistical differences. DW, dry weight; TDW, total dry weight; HI, harvest index.

The assessment of physiological parameters among various triticale cultivars reported clear differences, indicative of genotype-specific physiological attributes. For instance, triticale cv. C1 (PI388678) displayed the highest flavonoid concentration among the examined cultivars. Conversely, triticale Jordan, originating from Syria (C3) showed the lowest NDVI (1 and 2) readings and the highest anthocyanin concentration. Upon exposure to higher salinity levels, clear trends emerged in physiological parameters, highlighting the effects of salinity stress. Specifically, NDVI (1 and 2) readings, and δ^15^N exhibited a decreasing trend, indicative of salinity-induced stress. In contrast, N and C concentrations increased under saline conditions. Analysis of irrigation rates revealed differential impacts on physiological parameters. The lowest irrigation rate (I100) was linked with reduced NDVI 1 readings, coupled with elevated N and C concentration, δ^13^C, and nitrogen balance index (NBI) values. In contrast, the highest irrigation rate (I200) resulted in elevated flavonoids and anthocyanin concentrations. Furthermore, an examination of planting density highlighted differential trends in physiological parameters. Higher planting densities were associated with increased NDVI (1 and 2) readings, N and C concentrations. However, this increase in density was accompanied by a decrease in chlorophyll concentration (Chl) and δ^15^N (**Table 4**).

**Table 4:** Effects of cultivar (C1:; C2:; C3): salinity levels [S1: low electrical conductivity (EC) (1-2 dS m^-1^), S2: high EC (8-10 dS m^-1^)], irrigation levels (I1: 100% ET_0_, I2: 150% ET_0_ and I3: 200% ET_0_), and planting density (D1: 30 cm and D2: 50 cm of space between rows) on physiological parameters in triticale plants over the experimental period.

Factors			NDVI1 (-)	NDVI2 (-)	N concentration (%)	δ^15^N (‰)	C concentration (%)	δ^13^C (‰)	Chl (µg cm^-^²)	Flav (-)	Anth (-)	NBI (-)
**A**	**Cultivar**	**C1**	0.32 ± 0.08 ^a^	0.38 ± 0.09 ^a^	4.43 ± 0.65 _a_	1.78 ± 4.02 ^a^	44.03 ± 0.97 ^a^	-28.55 ± 0.89 ^a^	31.44 ± 6.78 ^a^	1.26 ± 0.19 ^a^	0.11 ± 0.03 ^c^	25.83 ± 5.24 ^a^
		**C2**	0.33 ± 0.09 ^a^	0.40 ± 0.10 ^a^	4.28 ± 0.80 _a_	1.53 ± 4.00 ^a^	43.96 ± 1.12 ^a^	-28.08 ± 2.47 ^a^	30.60 ± 6.86 ^a^	1.17 ± 0.22 ^b^	0.12 ± 0.02 ^b^	27.29 ± 5.84 ^a^
		**C3**	0.30 ± 0.08 ^b^	0.35 ± 0.10 ^b^	4.61 ± 0.61 _a_	1.39 ± 3.89 ^a^	43.97 ± 1.27 ^a^	-28.07 ± 1.15 ^a^	29.24 ± 7.90 ^a^	1.12 ± 0.17 ^b^	0.13 ± 0.04 ^a^	26.94 ± 6.15 ^a^
**B**	**Salinity**	**S1**	0.34 ± 0.08 ^a^	0.43 ± 0.09 ^a^	4.26 ± 0.70 ^b^	2.46 ± 4.62 ^a^	43.78 ± 1.26 ^b^	-28.49 ± 2.18 ^a^	29.85 ± 6.56 ^a^	1.17 ± 0.19 ^a^	0.12 ± 0.03 ^a^	26.42 ± 5.61 ^a^
		**S2**	0.30 ± 0.09 ^b^	0.34 ± 0.08 ^b^	4.60 ± 0.67 ^a^	0.71 ± 2.92 ^b^	44.18 ± 0.90 ^a^	-28.02 ± 0.93 ^a^	31.04 ± 7.83 ^a^	1.19 ± 0.21 ^a^	0.12 ± 0.03 ^a^	26.98 ± 5.90 ^a^
**C**	**Irrigation**	**I100**	0.30 ± 0.08 ^b^	0.39 ± 0.10 ^a^	4.80 ± 0.66 ^a^	-0.53 ± 3.28 ^c^	44.67 ± 0.85 ^a^	-27.74 ± 2.43 ^a^	31.65 ± 6.96 ^a^	1.13 ± 0.19 ^b^	0.12 ± 0.03 ^ab^	29.30 ± 6.41 ^a^
		**I150**	0.33 ± 0.09 ^a^	0.40 ± 0.11 ^a^	4.39 ± 0.47 ^b^	1.10 ± 2.94 ^b^	44.24 ± 0.92 ^b^	-28.20 ± 0.95 ^ab^	29.60 ± 7.48 ^a^	1.19 ± 0.20 ^ab^	0.12 ± 0.03 ^b^	25.41 ± 4.50 ^b^
		**I200**	0.32 ± 0.08 ^a^	0.36 ± 0.08 ^b^	4.09 ± 0.78 ^c^	4.12 ± 4.07 ^a^	43.07 ± 0.85 ^c^	-28.79 ± 1.18 ^b^	30.14 ± 7.14 ^a^	1.22 ± 0.21 ^a^	0.13 ± 0.04 ^a^	25.58 ± 5.53 ^b^
**D**	**Planting density**	**D30**	0.26 ± 0.05 ^b^	0.34 ± 0.08 ^b^	4.22 ± 0.75 ^b^	4.04 ± 3.31 ^a^	43.76 ± 1.01 ^b^	-27.99 ± 2.28 ^a^	31.34 ± 6.49 ^a^	1.19 ± 0.19 ^a^	0.12 ± 0.03 ^a^	27.35 ± 5.32 ^a^
		**D50**	0.36 ± 0.08 ^a^	0.43 ± 0.09 ^a^	4.61 ± 0.62 ^a^	-0.56 ± 3.12 ^b^	44.18 ± 1.15 ^a^	-28.47 ± 0.84 ^a^	29.46 ± 7.81 ^b^	1.18 ± 0.22 ^a^	0.13 ± 0.04 ^a^	25.99 ± 6.11 ^a^
Interaction												
**AB**			ns	ns	ns	ns	ns	ns	*	ns	ns	ns
**AC**			ns	ns	ns	ns	ns	ns	*	*	*	ns
**AD**			ns	ns	ns	ns	ns	ns	ns	ns	*	ns
**BC**			*	*	ns	*	ns	ns	ns	ns	*	ns
**BD**			*	*	*	*	*	ns	ns	*	*	*
**CD**			*	*	*	ns	*	ns	ns	ns	ns	ns
**ABC**			ns	ns	ns	ns	ns	ns	*	*	*	ns
**ABD**			ns	ns	ns	ns	ns	ns	ns	ns	ns	ns
**ACD**			ns	ns	ns	ns	ns	ns	ns	ns	ns	ns
**BCD**			*	*	*	ns	*	*	ns	*	*	*
**ABCD**			ns	ns	ns	ns	ns	ns	*	*	*	ns

Treatment values are the means ± standard deviation of 3 replicates per treatment. Values with different letters within a column are significantly different at p< 0.05 (analysis of variance and least significant difference test). ns, indicates non-statistical differences. * indicates statistical differences.

**Figure 2:**
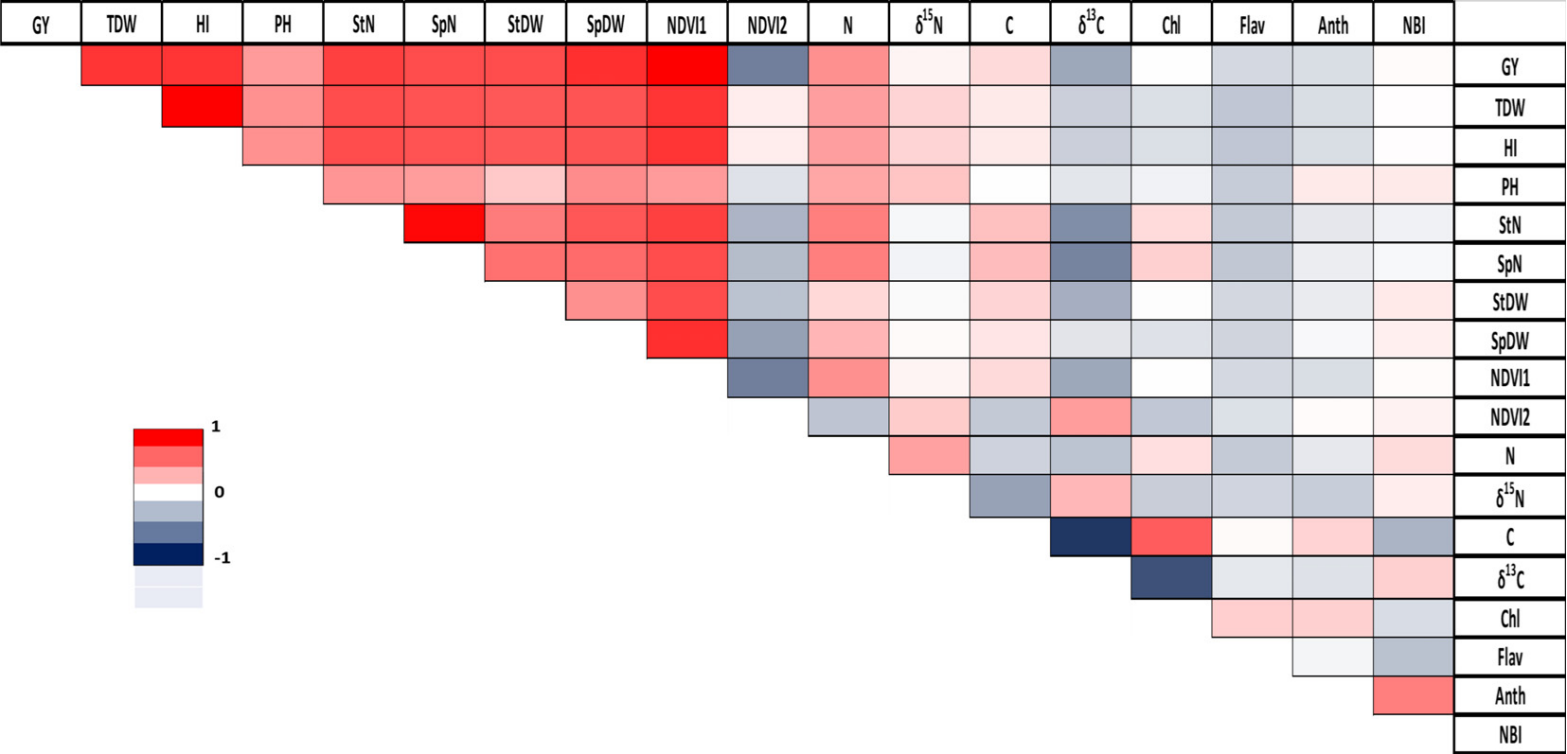
Correlation between agronomic and physiological parameters in triticale. GY, grain yield; TDW, total plant dry weight; HI, harvest index; PH, plant height; StN, stem number; SpN, spike number; StDW, stem dry weight; SpDW, spike dry weight; NDVI1, greenseeker reading 1; NDVI2, greenseeker reading 2; N, total nitrogen; δ^15^N, stable nitrogen isotope composition; C, total carbon; δ^13^C, stable carbon isotope composition; Chl, Chlorophyll; Flav, flavonoid; Anth, anthocyanin; NBI, Nitrogen Balance Index. Pearson correlation coefficient (r) is represented using a color gradient, where deep red corresponds to r=1, white indicates r=0, and deep blue represents r=-1.

The analysis of correlations across all triticale genotypes revealed significant positive correlations in total plant dry weight/harvest index (r = 1), grain yield/NDVI reading 1 (r = 1), and spike number/stem number (r = 0.97). Conversely, the strongest negative correlation was observed between C concentration/δ^13^C with a correlation coefficient of r = -0.76 (**Figure 2**). A more detailed analysis for each genotype is provided in the **Supplementary Material** (**Supplementary Figures S5–S7**).

### Effects of salinity, irrigation, and planting density levels on agronomic components, and physiological parameters in finger millet cultivars

The evaluation of agronomic parameters across distinct finger millet cultivars did not reveal significant differences in the parameters assessed. However, under increasing salinity levels, a consistent trend emerged wherein plant height, spike dry weight, and harvest index decreased, while stem dry weight increased. Analysis of irrigation rates revealed that the lowest irrigation rate (I100) resulted in the highest spike dry weight; however, it was also associated with the lowest values for both stem dry weight and harvest index. Furthermore, an exploration of planting density indicated a general augmentation across all parameters studied, except for the harvest index, which decreased with higher planting density (**Table 5**).

**Table 5:** Effects of cultivar (C1:; C2): salinity levels [S1: low electrical conductivity (EC) (1-2 dS m^-1^), S2: high EC (8-10 dS m^-1^)], irrigation levels (I1: 100% ET_0_, I2: 150% ET_0_ and I3: 200% ET_0_), and planting density (D1: 30 cm and D2: 50 cm of space between rows) on biomass and yield parameters in finger millet plants over the experimental period.

Factors			Plant height (cm)	Stem number per m^2^	Spike number per m^2^	Stem DW per m^2^	Spike DW per m^2^	Grain yield (kg ha^-1^)	TDW (kg ha^-1^)	HI (-)
**A**	**Cultivar**	**C1**	114.64 ± 12.78 ^a^	181.69 ± 60.18 ^a^	541.00 ± 219.57 ^a^	955.80 ± 450.70 ^a^	475.33 ± 303.52 ^a^	3216.02 ± 1042.71 ^a^	10333.13 ± 5178.92 ^a^	0.35 ± 0.12 ^a^
		**C2**	114.04 ± 16.39 ^a^	205.90 ± 124.25 ^a^	505.53 ± 193.51 ^a^	1030.49 ± 509.12 ^a^	414.25 ± 166.26 ^a^	3546.94 ± 1251.16 ^a^	10605.49 ± 5191.00 ^a^	0.38 ± 0.14 ^a^
**B**	**Salinity**	**S1**	117.97 ± 17.30 ^a^	197.17 ± 74.61 ^a^	483.75 ± 197.07 ^a^	825.02 ± 490.65 ^b^	534.86 ± 308.55 ^a^	3566.85 ± 1335.14 ^a^	10411.88 ± 5148.39 ^a^	0.38 ± 0.16 ^a^
		**S2**	110.71 ± 10.27 ^b^	190.43 ± 117.35 ^a^	562.78 ± 210.37 ^a^	1161.26 ± 407.82 ^a^	354.72 ± 98.98 ^b^	3196.11 ± 925.02 ^a^	10526.74 ± 5224.29 ^a^	0.34 ± 0.10 ^b^
**C**	**Irrigation**	**I100**	115.54 ± 17.81 ^a^	207.08 ± 146.09 ^a^	543.42 ± 222.81 ^a^	757.59 ± 470.55 ^b^	559.17 ± 236.25 ^a^	3033.61 ± 1209.78 ^a^	10260.22 ± 3157.28 ^a^	0.30 ± 0.07 ^b^
		**I150**	114.60 ± 14.15 ^a^	181.65 ± 61.30 ^a^	514.13 ± 190.68 ^a^	1017.73 ± 398.97 ^a^	408.38 ± 317.62 ^b^	3470.01 ± 1176.76 ^a^	10177.81 ± 6243.85 ^a^	0.39 ± 0.12 ^a^
		**I200**	112.88 ± 11.68 ^a^	192.67 ± 63.42 ^a^	512.25 ± 211.40 ^a^	1204.10 ± 471.54 ^a^	366.83 ± 84.28 ^b^	3640.83 ± 1034.09 ^a^	10969.90 ± 5703.40 ^a^	0.39 ± 0.16 ^a^
**D**	**Planting density**	**D30**	114.06 ± 14.41 ^a^	150.06 ± 39.29 ^b^	409.94 ± 152.67 ^b^	818.22 ± 359.84 ^b^	364.28 ± 194.97 ^b^	2774.44 ± 730.72 ^b^	6827.22 ± 1848.11 ^b^	0.43 ± 0.13 ^a^
		**D50**	114.63 ± 14.98 ^a^	237.54 ± 117.82 ^a^	636.58 ± 191.26 ^a^	1168.06 ± 522.28 ^a^	525.31 ± 265.21 ^a^	3988.52 ± 1190.36 ^a^	14111.39 ± 4806.54 ^a^	0.29 ± 0.08 ^b^
Interaction										
**AB**			ns	ns	ns	ns	ns	ns	ns	ns
**AC**			ns	ns	ns	ns	ns	ns	ns	ns
**AD**			ns	ns	ns	ns	ns	ns	ns	ns
**BC**			ns	ns	ns	*	*	ns	ns	*
**BD**			ns	ns	ns	ns	ns	ns	ns	ns
**CD**			ns	ns	ns	ns	*	ns	*	*
**ABC**			ns	ns	ns	ns	ns	ns	ns	ns
**ABD**			ns	ns	ns	ns	ns	ns	ns	ns
**ACD**			ns	ns	ns	ns	ns	ns	ns	ns
**BCD**			ns	ns	ns	ns	*	ns	ns	*
**ABCD**			ns	ns	ns	ns	ns	ns	ns	ns

Treatment values are the means ± standard deviation of 3 replicates per treatment. Values with different letters within a column are significantly different at p< 0.05 (analysis of variance and least significant difference test). ns, indicates non-statistical differences. * indicates statistical differences. DW, dry weight; TDW, total dry weight; HI, harvest index.

The assessment of physiological parameters across different genotypes of finger millet revealed distinct genotype-specific differences, highlighting the unique physiological attributes of each cultivar. Notably, finger millet cultivar 2 exhibited the highest concentrations of chlorophyll (Chl) and flavonoids (Flav), while showed the lowest levels of anthocyanins (Anth) among the two tested cultivars. As salinity levels increased, NDVI (1 and 2) readings, and δ^15^N showed a significant reduction, while leaf nitrogen concentration increased. Analysis of irrigation regimes revealed different patterns on physiological parameters. The lowest irrigation rate (I100) was associated with elevated N concentration, Chl concentration, Flav concentration, and nitrogen balance index (NBI). On the other hand, the highest irrigation rate (I200) led to decreased NBI values and increased δ^15^N and Anth concentrations. Furthermore, an examination of planting density elucidated clear trends in physiological parameters. Higher planting densities were associated with increased NDVI 1 readings, while concurrently resulting in decreased δ^15^N (**Table 6**).

**Table 6:** Effects of cultivar (C1:; C2): salinity levels [S1: low electrical conductivity (EC) (1-2 dS m^-1^), S2: high EC (8-10 dS m^-1^)], irrigation levels (I1: 100% ET_0_, I2: 150% ET_0_ and I3: 200% ET_0_), and planting density (D1: 30 cm and D2: 50 cm of space between rows) on physiological parameters in finger millet plants over the experimental period.

Factors			NDVI1 (-)	NDVI2 (-)	N concentration (%)	δ^15^N (‰)	C concentration (%)	δ^13^C (‰)	Chl (µg cm^-^²)	Flav (-)	Anth (-)	NBI (-)
**A**	**Cultivar**	**C1**	0.31 ± 0.07 ^a^	0.42 ± 0.08 ^a^	2.78 ± 0.46 ^a^	4.38 ± 2.97 ^a^	42.79 ± 0.74 ^a^	-14.33 ± 0.42 ^a^	32.71 ± 7.24 ^b^	1.24 ± 0.16 ^b^	0.12 ± 0.03 ^a^	26.91 ± 4.81 ^a^
		**C2**	0.30 ± 0.07 ^a^	0.43 ± 0.08 ^a^	2.76 ± 0.43 ^a^	5.14 ± 2.34 ^a^	42.63 ± 0.83 ^a^	-13.42 ± 4.79 ^a^	35.87 ± 4.39 ^a^	1.32 ± 0.10 ^a^	0.11 ± 0.02 ^b^	28.07 ± 4.10 ^a^
**B**	**Salinity**	**S1**	0.33 ± 0.06 ^a^	0.46 ± 0.08 ^a^	2.66 ± 0.37 ^b^	5.30 ± 2.83 ^a^	40.49 ± 0.94 ^a^	-14.19 ± 0.52 ^a^	33.62 ± 6.14 ^a^	1.26 ± 0.12 ^a^	0.11 ± 0.03 ^a^	27.43 ± 4.72 ^a^
		**S2**	0.28 ± 0.07 ^b^	0.37 ± 0.06 ^b^	2.88 ± 0.48 ^a^	4.25 ± 2.45 ^b^	41.52 ± 0.77 ^a^	-13.58 ± 4.73 ^a^	34.99 ± 6.18 ^a^	1.30 ± 0.15 ^a^	0.11 ± 0.03 ^a^	27.55 ± 4.28 ^a^
**C**	**Irrigation**	**I100**	0.29 ± 0.07 ^a^	0.42 ± 0.09 ^a^	3.05 ± 0.40 ^a^	3.47 ± 2.60 ^b^	43.19 ± 0.41 ^a^	-14.34 ± 0.41 ^a^	37.31 ± 3.31 ^a^	1.34 ± 0.10 ^a^	0.10 ± 0.01 ^b^	29.03 ± 3.30 ^a^
		**I150**	0.31 ± 0.06 ^a^	0.44 ± 0.09 ^a^	2.73 ± 0.25 ^b^	4.41 ± 2.13 ^b^	42.70 ± 0.71 ^a^	-14.11 ± 0.25 ^a^	34.58 ± 5.63 ^b^	1.26 ± 0.12 ^b^	0.10 ± 0.02 ^b^	27.95 ± 3.84 ^b^
		**I200**	0.31 ± 0.07 ^a^	0.40 ± 0.06 ^a^	2.57 ± 0.50 ^b^	6.29 ± 2.58 ^a^	42.58 ± 0.78 ^a^	-13.21 ± 5.82 ^a^	31.23 ± 7.30 ^c^	1.24 ± 0.16 ^b^	0.13 ± 0.04 ^a^	25.62 ± 5.41 ^c^
**D**	**Planting density**	**D30**	0.28 ± 0.05 ^b^	0.41 ± 0.08 ^a^	2.78 ± 0.48 ^a^	6.31 ± 1.87 ^a^	42.70 ± 0.88 ^a^	-13.35 ± 4.84 ^a^	34.74 ± 5.92 ^a^	1.30 ± 0.14 ^a^	0.11 ± 0.03 ^a^	27.62 ± 4.69 ^a^
		**D50**	0.33 ± 0.07 ^a^	0.43 ± 0.09 ^a^	2.77 ± 0.40 ^a^	3.30 ± 2.52 ^b^	42.72 ± 0.68 ^a^	-14.37 ± 0.51 ^a^	33.81 ± 6.44 ^a^	1.26 ± 0.13 ^a^	0.12 ± 0.03 ^a^	27.35 ± 4.30 ^a^
Interaction												
**AB**			ns	ns	ns	ns	ns	ns	ns	ns	ns	ns
**AC**			*	ns	ns	ns	ns	ns	ns	ns	ns	ns
**AD**			ns	ns	ns	ns	ns	ns	ns	ns	*	ns
**BC**			*	*	ns	ns	ns	ns	*	*	ns	*
**BD**			ns	ns	*	*	ns	ns	*	ns	*	*
**CD**			ns	*	ns	ns	ns	ns	*	*	*	ns
**ABC**			ns	ns	ns	ns	ns	ns	*	ns	*	*
**ABD**			ns	ns	ns	ns	ns	ns	ns	ns	ns	ns
**ACD**			ns	*	ns	ns	ns	ns	ns	ns	ns	ns
**BCD**			*	*	*	*	ns	ns	*	ns	*	*
**ABCD**			ns	ns	ns	ns	ns	ns	*	*	*	ns

Treatment values are the means ± standard deviation of 3 replicates per treatment. Values with different letters within a column are significantly different at p< 0.05 (analysis of variance and least significant difference test). ns, indicates non-statistical differences. * indicates statistical differences.

**Figure 3:**
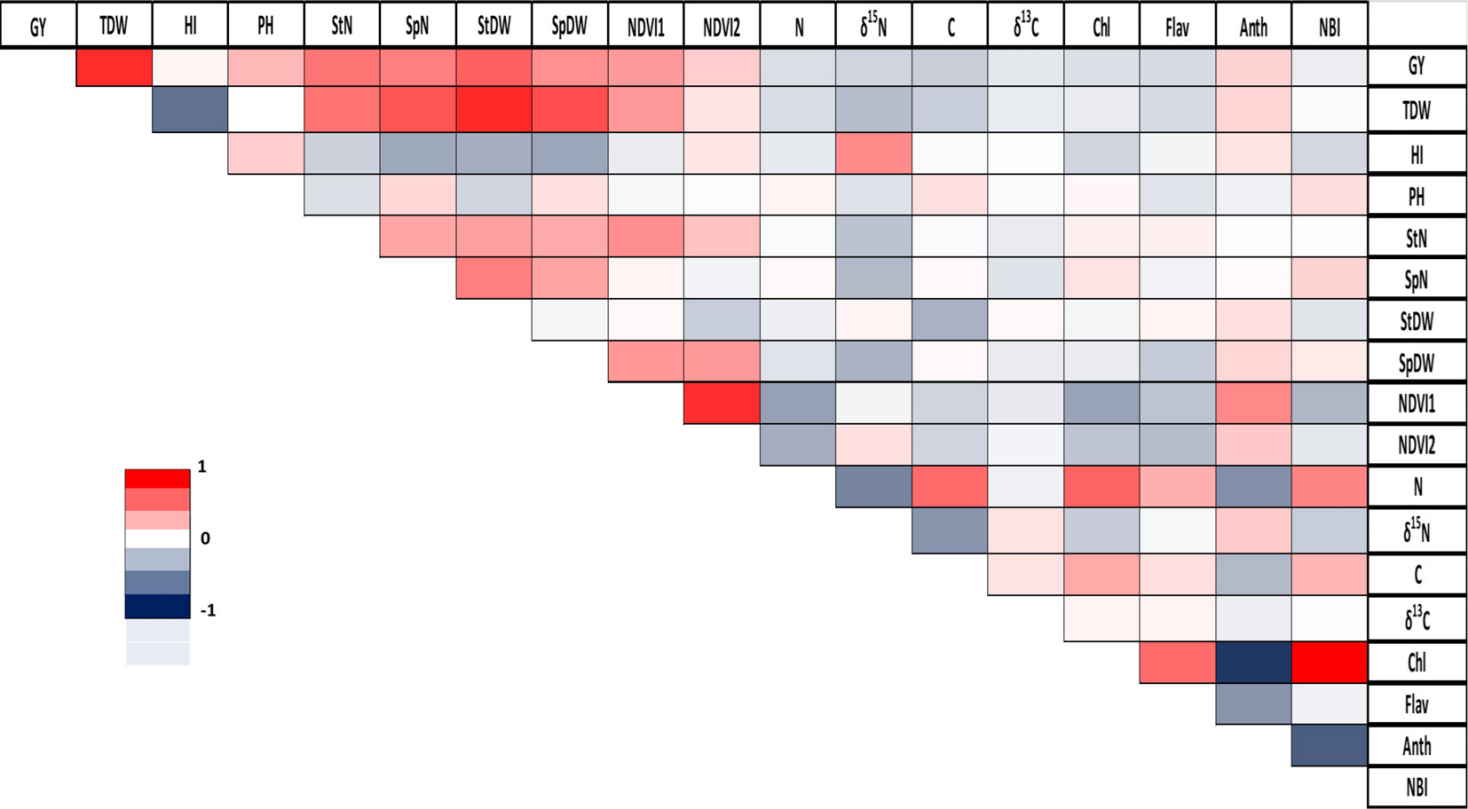
Correlation between agronomic and physiological parameters in finger millet. GY, grain yield; TDW, total plant dry weight; HI, harvest index; PH, plant height; StN, stem number; SpN, spike number; StDW, stem dry weight; SpDW, spike dry weight; NDVI1, greenseeker reading 1; NDVI2, greenseeker reading 2; N, total nitrogen; δ^15^N, stable nitrogen isotope composition; C, total carbon; δ^13^C, stable carbon isotope composition; Chl, Chlorophyll; Flav, flavonoid; Anth, anthocyanin; and NBI, Nitrogen Balance Index. Pearson correlation coefficient (r) is represented using a color gradient, where deep red corresponds to r=1, white indicates r=0, and deep blue represents r=-1.

The analysis of correlations across all finger millet genotypes reveals significant positive correlations in Chl/NBI (r = 0.83) and density/total plant dry weight (r = 0.72). Conversely, the strongest negative correlation was observed between Chl/Anth with a correlation coefficient of r = -0.88 (**Figure 3**). A more detailed analysis for each genotype is provided in the **Supplementary Material** (**Supplementary Figures S8, S9**).

**Figure 4:**
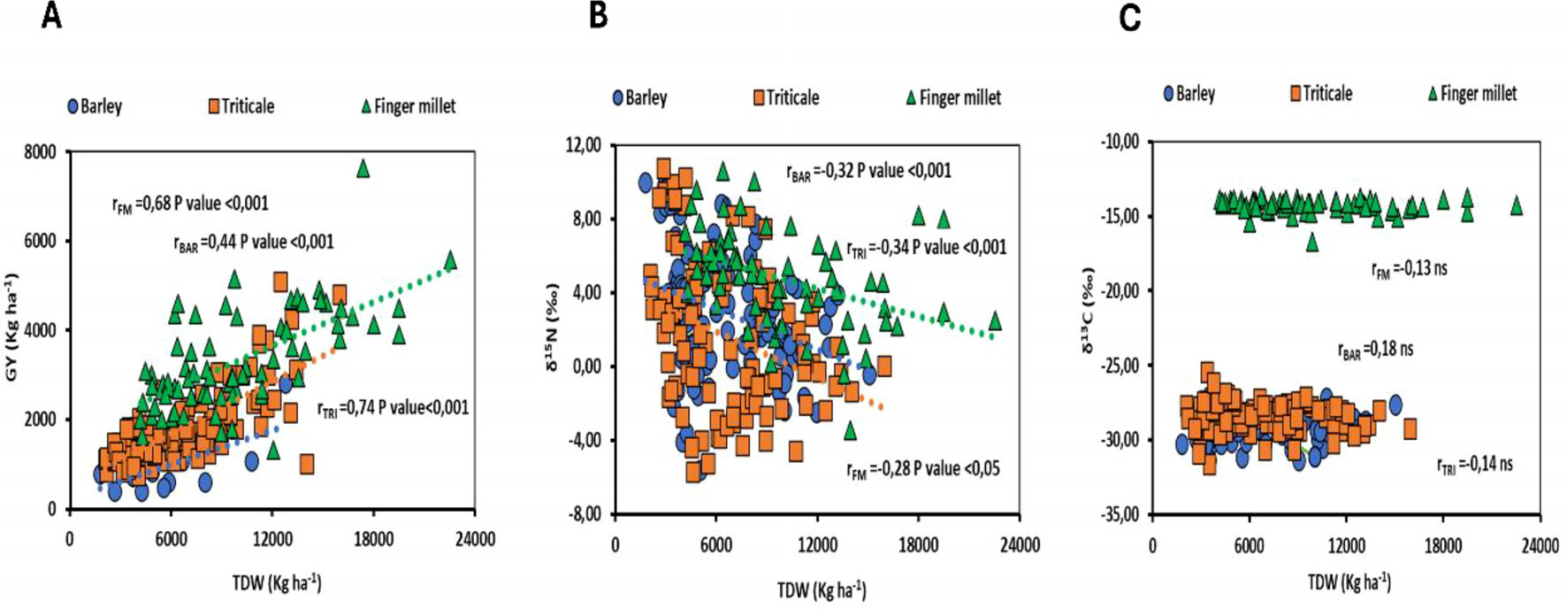
**(A)** Relationship between grain yield (GY) and total plant dry weight (TDW), **(B)** Relationship between nitrogen isotope composition (δ^15^N) and total plant dry weight (TDW) and **(C)** Relationship between carbon isotope composition (δ^13^C) and total plant dry weight (TDW). The figures presented encompass data from all genotypes, analyzed across varying levels of salinity, irrigation, and planting density within the studied species. Statistical significance at *P*<0.001, *P*<0.01, *P*<0.05 and ns (non-statistically significant).

The analysis of the correlation between total plant dry weight (TDW) and grain yield (GY) revealed a strong positive relationship across all species examined, with triticale displaying the highest correlation value (r = 0.74). Furthermore, the study identified a negative correlation between nitrogen isotope composition and total plant dry weight in both barley and triticale. Conversely, no significant correlation was observed between carbon isotope composition and total plant dry weight across the species studied (**Figure 4**).

## Discussion

### Effects of agronomic practices and genotypes in barley yield and physiology parameters

There were clear genotypic effects on agronomic traits evaluated in barley plants. Among the cultivars evaluated, N2-35 demonstrated superior agronomic performance, particularly in terms of grain yield (GY) and total plant dry weight (TDW). In our experiment, increasing salinity levels did not affect any of the agronomic parameters assessed in barley. Contrarily, other researchers have reported a significant decline in several agronomic parameters such as the number of spikes per m^2^ and grain yield under saline conditions (Hessini et al., 2015). Such discrepancies can likely be attributed to variations in the electrical conductivity of irrigation water used and the drainage capacities of the soil in which the experiments were conducted. In contrast, water deficit in our study resulted in a notable reduction in grain yield. Although barley is considered one of the most resilient cereal crops under moderate water deficit conditions (Robredo et al., 2007; Sanchez-Díaz et al., 2002), water stress has been shown to significantly reduce yield (Bahadur et al., 2013; Shrief and Abd-El-Mohsen, 2014; Pardo et al., 2022). It is important to note that, beyond water availability, other factors such as vapor pressure deficit (VPD), temperature and radiation can also affect yield performance in barley and other cereal crops (Van Ittersum et al., 2013; Dreccer et al., 2018). Both water deficit and salt stress impose detrimental effects on grain yield by reducing photosynthesis, restricting cell growth, limiting leaf expansion, and decreasing transpiration (Sabagh et al., 2019). In our experiment, heightened planting densities were associated with an overall improvement across agronomic parameters, consistent with findings from previous studies (Soleymani et al., 2011). Nevertheless, contrasting trends have been reported in other studies (Soleymani and Shahrajabian, 2011; Ertekin, 2022), highlighting the complex interplay of factors influencing barley performance under varying environmental and management conditions.

From a physiological perspective, the growth of barley plants under saline conditions resulted in increased δ¹³C values, consistent with findings previously reported by Araus et al. (2021). This enrichment in δ¹³C is likely associated with stomatal closure, which reduces the ratio of intercellular to atmospheric CO_2_ concentration (Ci/Ca) (Munns and Tester, 2008). Furthermore, this phenomenon can be directly associated with the activity of Rubisco, the primary enzyme in carbon fixation, which exhibits altered discrimination against ^13^C under conditions of reduced CO_2_ availability (Abdulbaki et al., 2022). In addition, the observed increases in total chlorophyll and NBI may be attributed to the effects of salinity, which induces the development of thicker or more compact leaves. This response, however, may be interpreted as a negative reaction to stress, as similarly observed in quinoa plants by Rezzouk et al. (2020b). Anthocyanin levels also increased under salt stress, consistent with the findings reported by Mansour (2023). Moreover, the reduction in nitrogen isotope composition values observed under saline conditions may be attributed to the well-documented antagonism between chloride and nitrate (Corrado et al., 2020). This reduction in nitrogen concentration, which can adversely impact crop yield due to its critical role in plant growth and development can be mitigated with the application of nitrogenous fertilizers.

Barley plants exposed to the lowest irrigation levels exhibited reduced NDVI readings (1 and 2) alongside an increase in carbon concentration. The observed increase in carbon (C) concentration under water stress conditions in our experiment aligns with the findings reported by Rezzouk et al. (2022). The reduction in NDVI readings under water stress is likely due to its negative impact on green aboveground biomass (AB), as previously documented by Aparicio et al. (2000, 2004). The assessment of aboveground biomass (AB) is crucial for monitoring crop growth, as it can indicate the effects of various stresses on crop development (Araus et al., 2008; Elazab et al., 2015).

The observed increases in NDVI readings, chlorophyll concentration, nitrogen concentration, and Nitrogen Balance Index (NBI) in response to higher planting densities can be interpreted as a compensatory mechanism to mitigate the effects of reduced light distribution within the crop canopy (Ming et al., 2017). This reduction in light interception likely stimulates the accumulation of photosynthetic pigments, enabling the plants to maintain optimal photosynthetic efficiency suboptimal light conditions. As a result, this adaptation is reflected in the intensified green coloration of the leaves and an elevated nitrogen content (Paradiso and Proietti, 2022). The strongest positive correlations between agronomic parameters, such as stem number/spike number as well as total plant dry weight/spike dry weight, are expected, as these traits are intrinsically linked to the successful progression of various growth stages in barley plants.

### Effects of agronomic practices and genotypes in triticale yield and physiology parameters

There were no significant genotypic effects on most of the agronomic traits assessed in triticale cultivars. Increasing electrical conductivity in the irrigation water resulted in a marked decline in agronomic parameters observed in triticale except for stem dry weight. These findings align with the established moderate tolerance of this species to salt stress and are consistent with previous research on its salinity responses (Karim et al., 1993; Mohammadi Alagoz et al., 2023). Triticale’s reduced performance under salinity stress arises from both physiological constraints (osmotic imbalance, ion toxicity, oxidative stress) and genetic limitations (weaker expression of salt-tolerance genes, incomplete inheritance of rye’s resilience) (Blum, 2014). The development of triticale cultivars with improved salt tolerance has the potential to significantly enhance this trait, thereby contributing to increasing yield. The highest agronomic performance was recorded in triticale plants subjected to an intermediate irrigation rate. This observation is consistent with prior literature, which highlights the positive impact of increased irrigation, particularly under arid conditions, on triticale agronomic performance (Yusuf et al., 2023). In addition, higher planting densities resulted in improvements across all assessed agronomic parameters, supporting findings from previous experiments on triticale under varying planting densities (Giunta and Motzo, 2004; Mendoza-Elos et al., 2011). However, it is important to note that these results may be influenced by season, cultivar, and site-specific factors. Enhanced agronomic performance under higher planting densities is beneficial, as it indicates that closely spaced plants have a better performance using available resources such as water and nutrients, moreover than incoming radiation, therefore maximizing land use efficiency.

From a physiological perspective, irrigation with saline water led to reductions in NDVI readings and δ^15^N values. The observed decline in NDVI readings is likely associated with reduced photosynthetic efficiency under saline conditions, which induces pigment photo-oxidation (Stefanov et al., 2021). Photo-oxidation can impair the functionality of the photosynthetic machinery, thereby affecting plant performance. Additionally, salinity disrupts nitrogen uptake, assimilation, release, and internal recycling processes, contributing to the observed decrease in plant δ^15^N (Cernusak et al., 2009), as evidenced in our experiment. The observed increase in nitrogen and carbon concentrations in triticale plants under salt stress may be attributed to the synthesis of carbon-rich secondary compounds, such as phenolics and lignin, as well as nitrogen-rich metabolites, including amino acids (Hurtado et al., 2020). These metabolic shifts are likely adaptive responses designed to mitigate the adverse effects of soil salinity. Furthermore, the observed increase in flavonoid and anthocyanin levels in leaves under the highest irrigation rate could be associated with the protective role that these compounds play in mitigating damage to photosystem II caused by excessive water irrigation (Agati et al., 2021; Sperdouli et al., 2021).

Higher planting densities in triticale plants were associated with increased NDVI (1 and 2) readings, as well as higher %N and %C content, reflecting enhanced physiological activity and resource acquisition within densely populated crop stands (Schulze and Chapin, 1987). However, this increase in density was accompanied by a decrease in chlorophyll concentration (Chl) and δ^15^N, highlighting a trade-off between resource utilization and physiological performance under intensified intra-specific competition (Auer et al., 2020). The reduced δ^15^N values suggest that diminished access to nitrogen sources, driven by interspecific competition, resulted in decreased nitrogen availability. This limitation adversely impacted chlorophyll synthesis, thereby resulting in a decline in chlorophyll concentration.

The strong positive correlations, with coefficients approaching 1, observed between total plant dry weight and harvest index, as well as between spike number and stem number, underscore the uniformity in growth patterns among triticale plants subjected to varying agronomic practices and genotypic differences.

### Effects of agronomic practices and genotypes in finger millet yield and physiology parameters

There were no clear genotypic effects on the agronomic traits evaluated in finger millet plants. Similar to triticale, certain agronomic parameters, such as plant height and spike dry weight, declined with increasing electrical conductivity in the irrigation water. These findings align with the results reported by Krishnamurthy et al. (2014), who evaluated the tolerance of different cultivars of finger millet under rising salinity levels in the soil solution. Notably, the lowest irrigation rate (I100) resulted in the highest spike dry weight in our experiment, aligning with the findings reported by Krishnamurthy et al. (2016) in their evaluation of a minicore collection of finger millet germplasm. Increasing planting density resulted in a clear increase across all agronomic parameters studied. However, contrasting results have been documented in other studies involving finger millets plants subjected to increased planting densities (Shinggu et al., 2009; Shinggu and Gani, 2012). Similar to triticale, the improved performance in using available resources such as water and nutrients, along with the maximization of land use efficiency, suggests that increasing planting density could be a valuable agronomic practice in arid regions.

From a physiological perspective, similar to triticale plants, finger millet plants subjected to increasing EC resulted in a reduction in NDVI readings and δ^15^N values. The decline in NDVI readings observed in this study is likely attributable to a reduction in photosynthetic efficiency under saline conditions, which promotes pigment photo-oxidation (Stefanov et al., 2021). Furthermore, salinity appears to disrupt key processes involved in nitrogen uptake, assimilation, release, and internal recycling, thereby contributing to the observed decrease in plant δ^15^N (Cernusak et al., 2009).

Finger millet plants subjected to the highest levels of irrigation exhibited decreased Nitrogen Balance Index (NBI) values, alongside increased δ^15^N and anthocyanin concentrations. Abiotic stresses, such as overirrigation, can disrupt nitrogen uptake, assimilation, and release processes, contributing to the observed increase in δ^15^N and corresponding decrease in NBI. This may be attributed to the accumulation or breakdown of free amino acids, which play a critical role in maintaining cellular homeostasis (Xu et al., 2005; Xu and Yu, 2006). Additionally, similar to plant responses under salt and drought stress, anthocyanin levels have been shown to rise in response to excessive irrigation (Chalker-Scott, 2002).

Finger millet plants cultivated at higher planting densities exhibited increased NDVI readings alongside reduced δ^15^N values. The increase in NDVI readings may reflect a compensatory response to reduced light distribution across the canopy, a common characteristic of crops grown under higher planting densities (Ming et al., 2017). Furthermore, the reduction in δ^15^N values is likely attributable to intensified competition for nutrients, particularly nitrogen, under higher planting densities, as supported by the findings of Ciampitti and Vyn (2011) and Dai et al. (2014). The δ¹^3^C values of finger millet were typical of a C4 species (Varalli et al., 2024), where the range of variability associated with water and salinity stresses far is lower than that of C3 plants (Farquhar et al., 1989).

Our findings revealed a strong positive correlation between grain yield (GY) and total plant dry weight (TDW) across the different species studied, which is consistent with the results reported by other researchers in cereal crops (Elazab et al., 2015; Rezzouk et al., 2022). The stable isotopic compositions of carbon and nitrogen proved to be valuable indicators for assessing crop responses to varying environmental conditions. In this context, we analyzed the relationships between these isotopic compositions and total plant dry weight across the different species studied, considering genotype-specific variations and the diverse agronomic practices employed. In our experiment, a negative correlation between nitrogen isotope composition and total plant dry weight was observed across all three species, indicating restricted nitrogen uptake under adverse conditions. This finding is consistent with expectations, as nitrogen uptake is commonly reduced under stressors such as salinity, drought, or increased planting density (Ehtaiwesh, 2022; He and Dijkstra, 2014; Zhang et al., 2016). In contrast, no significant relationship was identified between carbon isotope composition and total plant dry weight across the species studied, with correlation values generally low. Despite this, previous research has demonstrated that correlations between carbon isotope composition and total plant dry weight can be significant, with the direction of the correlation-either negative or positive-depending on the specific plant tissue sampled and the environmental conditions tested (Voltas et al., 1999). In fact, the combination of different planting densities, which strong affect total dry weight independently of the water stress, may justify the absence of such a correlation between δ¹³C and total plant dry weight. Additionally, phenology may significantly influence both total plant dry weight and carbon isotope composition, potentially accounting for observed negative relationships (Condon et al., 2004). Consequently, when investigating the relationship between δ¹³C and total plant dry weight (TDW), it is crucial to evaluate genotypes at comparable phenological stages (Richards et al., 2011).

## Conclusion

The results of this study demonstrate that genotypic variation among barley, triticale and finger millet accessions led to distinct trends in agronomic and physiological parameters in combinatorial stress treatment in arid regions. Additionally, the differential tolerance of these cereal species to salt, drought and planting densities significantly influenced the experimental outcomes. In barley, C1 (N2-35) showed superior biomass, while C2 (N2-4) had the highest harvest index. Low irrigation reduced yield, and higher planting densities improved most agronomics parameters but reduced harvest index. Salinity had minimal agronomical effects but affected physiological traits, increasing nitrogen, δ^13^C, chlorophyll and anthocyanins in the two C3 crop species. For triticale, cv. C2 (PI429152) showed the lowest grain yield and harvest index. Medium irrigation enhanced growth, while planting densities increased all agronomic parameters studied. Salinity stress reduced NDVI and δ¹^5^N, while nitrogen and carbon concentrations increased. In finger millet, salinity reduced growth and harvest index. Low irrigation increased spike dry weight but reduced other parameters. Higher planting densities improved most traits but reduced harvest index. While this experiment has provided valuable insights into the agronomic and physiological responses of three cereal species, further research is needed to enhance total aerial biomass and grain productivity in arid environments. The current study has suffered one limitation such as the lack of seasonal replication. Nevertheless, futures studies could address these limitations, including for example multiple planting cycles or a similar plating date, but across successive years, to provide a broader understanding of how environmental factors interact with the factors already studied.

## Data Availability

The original contributions presented in the study are included in the article/**Supplementary Material**. Further inquiries can be directed to the corresponding author.
