# Supplementary material for: Optimization of cereal productivity and physiological performance under desert conditions: varying irrigation, salinity and planting density levels
Source: Front Plant Sci. 2025 Mar 6;16:1488576. doi: 10.3389/fpls.2025.1488576 (PMC11922717; doi:10.3389/fpls.2025.1488576)

**Figure S1.** Daily mean temperature and rainfall pattern during the experimental period.

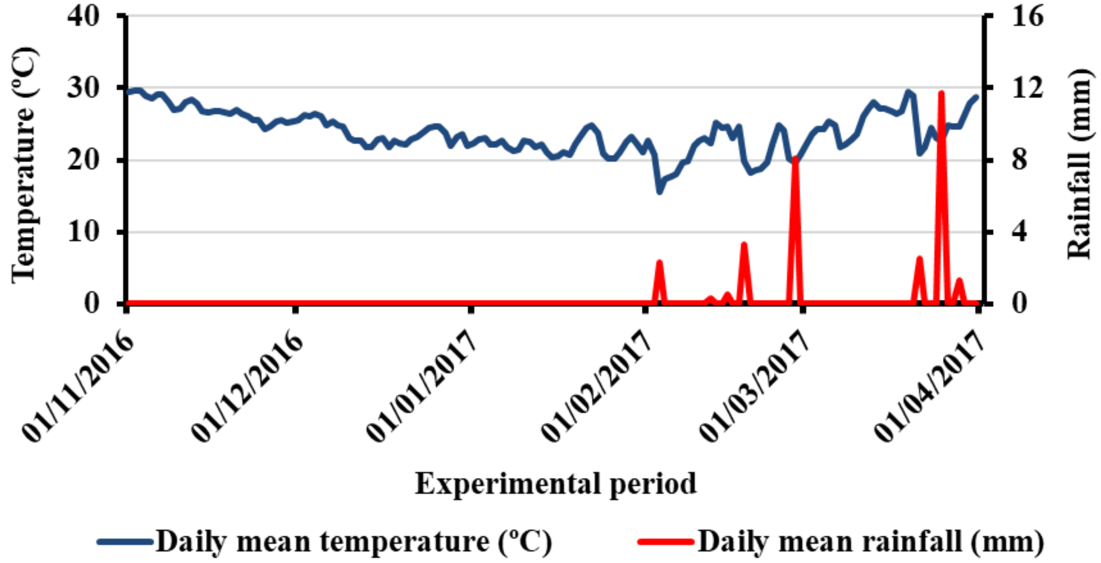

**Figure S2.** Relationships among agronomical and physiological parameters in barley cultivar N2-35. GY: grain yield, TDW: total plant dry weight, HI: harvest index, PH: plant height, StN: stem number, SpN: spike number, StDW: stem dry weight, SpDW: spike dry weight, NDVI1: greenseeker reading 1, NDVI2: greenseeker reading 2, N: total nitrogen, $\delta^{15}N$: stable nitrogen isotope composition, C: total carbon, $\delta^{13}C$: stable carbon isotope composition, Chl: Chlorophyll, Flav: flavonoid, Anth: anthocyanin, and NBI: Nitrogen Balance Index. Pearson correlation coefficient (r).

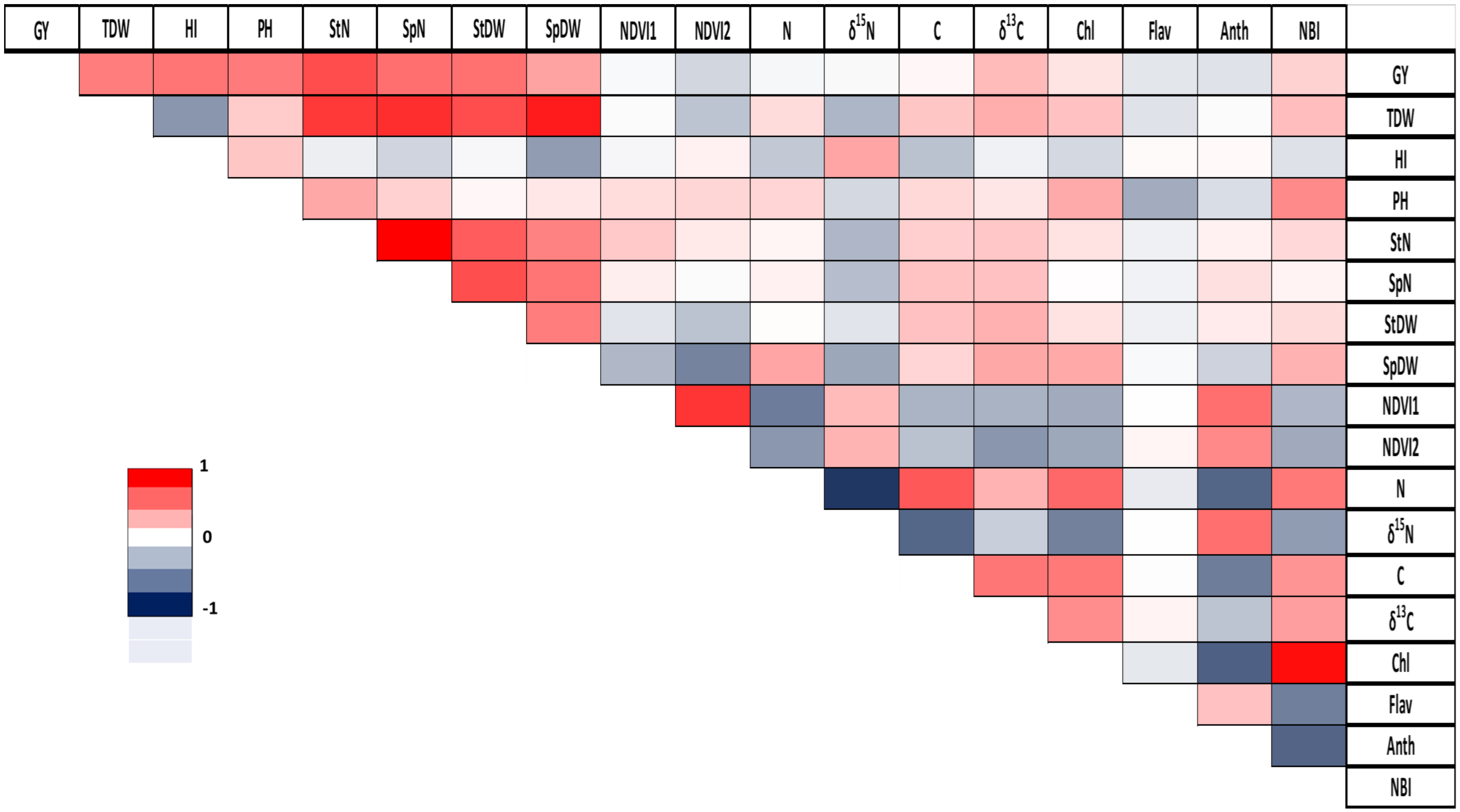

**Figure S3.** Relationships among agronomical and physiological parameters in barley cultivar N2-4. GY: grain yield, TDW: total plant dry weight, HI: harvest index, PH: plant height, StN: stem number, SpN: spike number, StDW: stem dry weight, SpDW: spike dry weight, NDVI1: greenseeker reading 1, NDVI2: greenseeker reading 2, N: total nitrogen, $\delta^{15}N$: stable nitrogen isotope composition, C: total carbon, $\delta^{13}C$: stable carbon isotope composition, Chl: Chlorophyll, Flav: flavonoid, Anth: anthocyanin, and NBI: Nitrogen Balance Index. Pearson correlation coefficient (r).

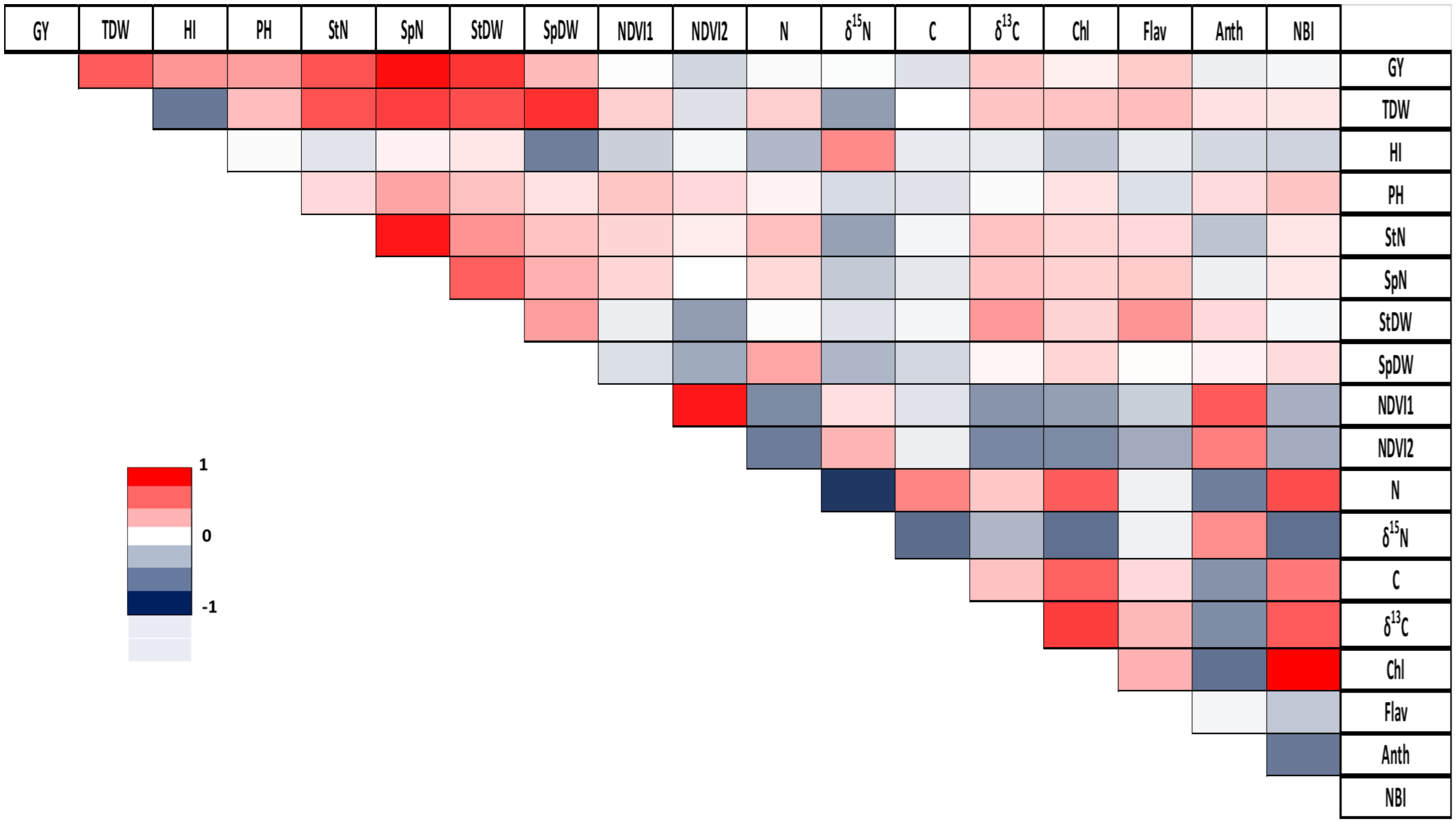

**Figure S4.** Relationships among agronomical and physiological parameters in barley cultivar IPA7. GY: grain yield, TDW: total plant dry weight, HI: harvest index, PH: plant height, StN: stem number, SpN: spike number, StDW: stem dry weight, SpDW: spike dry weight, NDVI1: greenseeker reading 1, NDVI2: greenseeker reading 2, N: total nitrogen, $\delta^{15}N$: stable nitrogen isotope composition, C: total carbon, $\delta^{13}C$: stable carbon isotope composition, Chl: Chlorophyll, Flav: flavonoid, Anth: anthocyanin, and NBI: Nitrogen Balance Index. Pearson correlation coefficient (r).

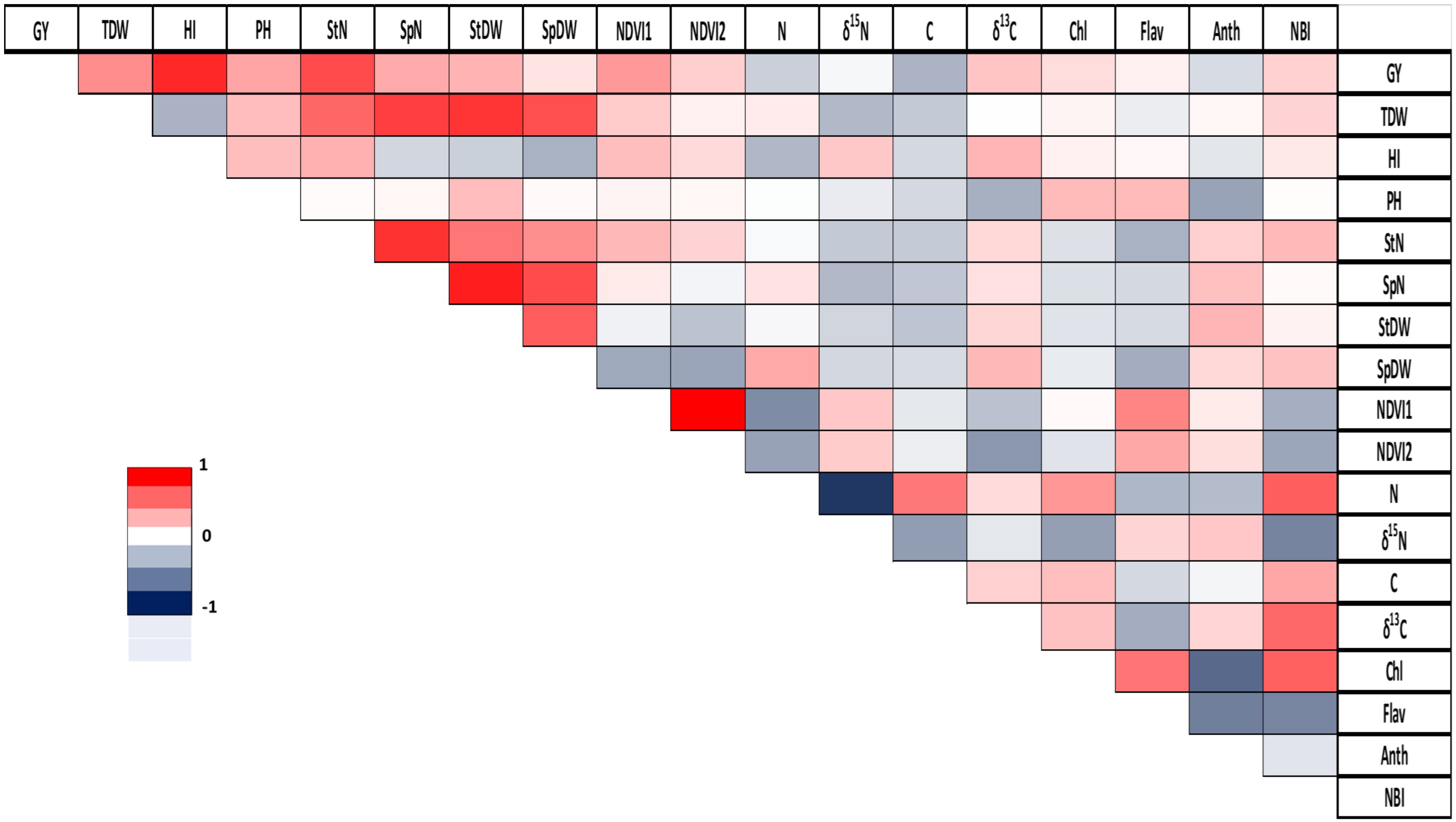

**Figure S5.** Relationships among agronomical and physiological parameters in triticale cultivar PI388678. GY: grain yield, TDW: total plant dry weight, HI: harvest index, PH: plant height, StN: stem number, SpN: spike number, StDW: stem dry weight, SpDW: spike dry weight, NDVI1: greenseeker reading 1, NDVI2: greenseeker reading 2, N: total nitrogen, $\delta^{15}N$: stable nitrogen isotope composition, C: total carbon, $\delta^{13}C$: stable carbon isotope composition, Chl: Chlorophyll, Flav: flavonoid, Anth: anthocyanin, and NBI: Nitrogen Balance Index. Pearson correlation coefficient (r).

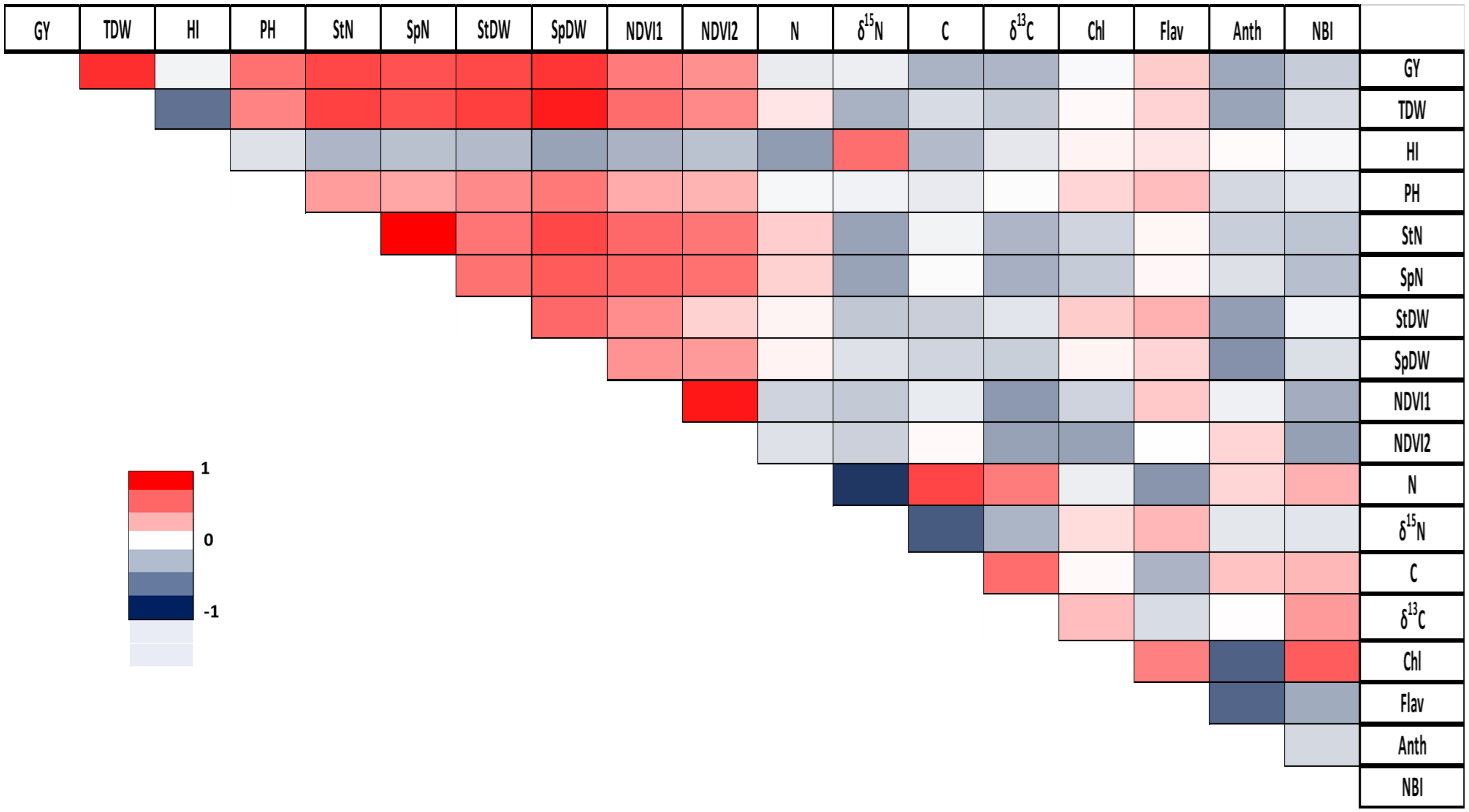

**Figure S6.** Relationships among agronomical and physiological parameters in triticale cultivar PI4295152. GY: grain yield, TDW: total plant dry weight, HI: harvest index, PH: plant height, StN: stem number, SpN: spike number, StDW: stem dry weight, SpDW: spike dry weight, NDVI1: greenseeker reading 1, NDVI2: greenseeker reading 2, N: total nitrogen, $\delta^{15}N$: stable nitrogen isotope composition, C: total carbon, $\delta^{13}C$: stable carbon isotope composition, Chl: Chlorophyll, Flav: flavonoid, Anth: anthocyanin, and NBI: Nitrogen Balance Index. Pearson correlation coefficient (r).

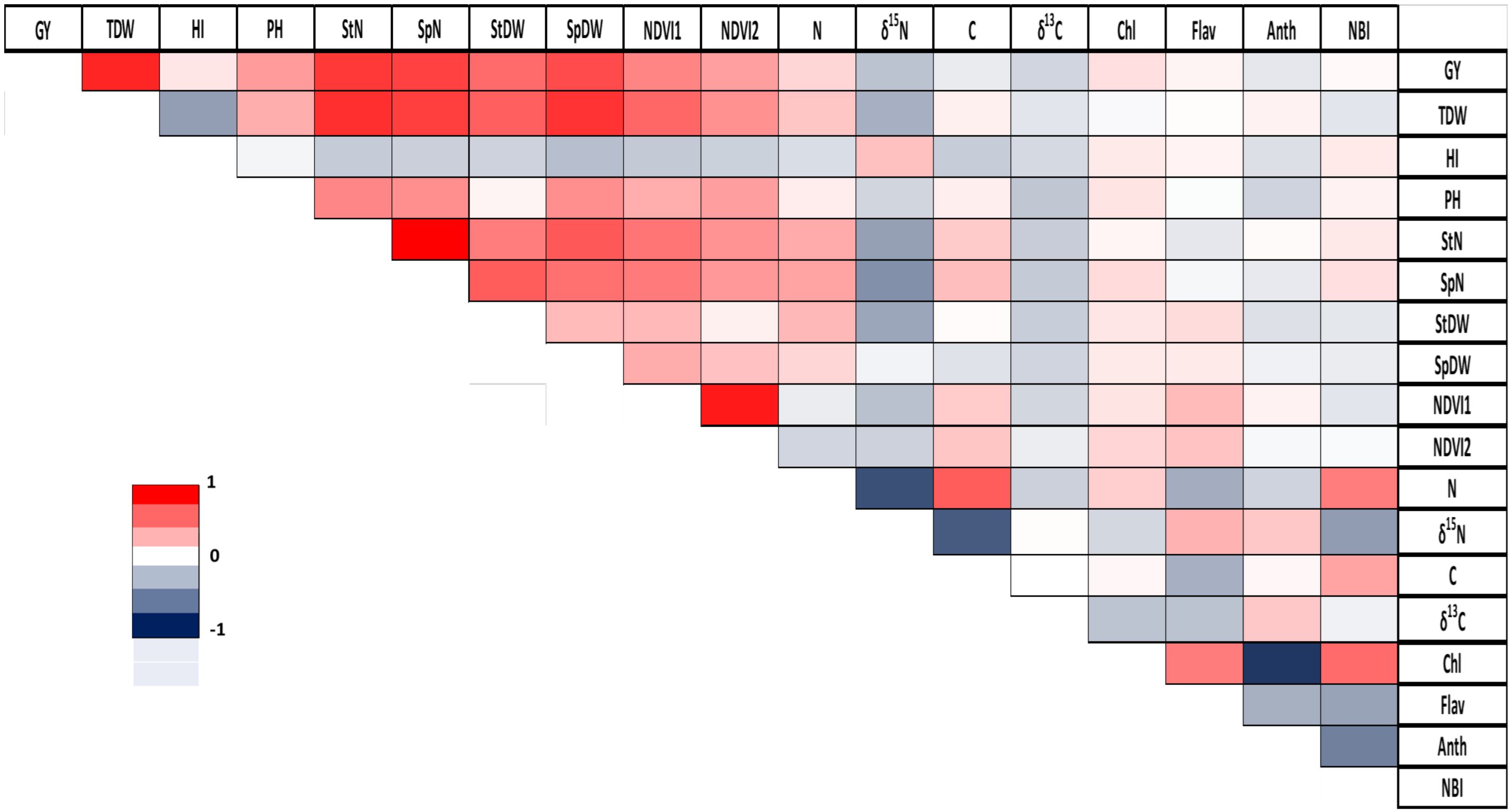

**Figure S7.** Relationships among agronomical and physiological parameters in triticale cultivar from Jordan but originally from Syria. GY: grain yield, TDW: total plant dry weight, HI: harvest index, PH: plant height, StN: stem number, SpN: spike number, StDW: stem dry weight, SpDW: spike dry weight, NDVI1: greenseeker reading 1, NDVI2: greenseeker reading 2, N: total nitrogen, $\delta^{15}N$: stable nitrogen isotope composition, C: total carbon, $\delta^{13}C$: stable carbon isotope composition, Chl: Chlorophyll, Flav: flavonoid, Anth: anthocyanin, and NBI: Nitrogen Balance Index. Pearson correlation coefficient (r).

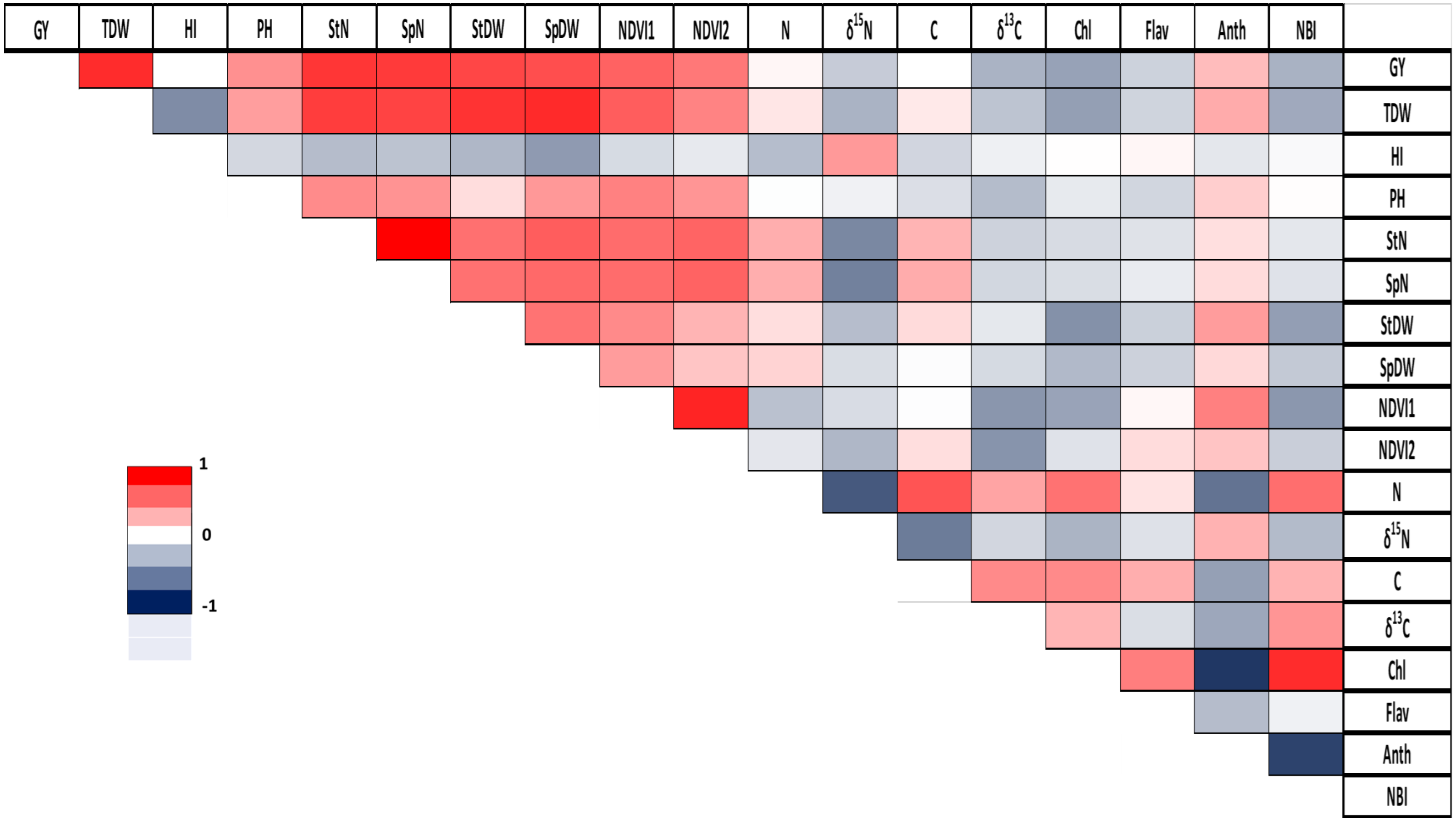

**Figure S8.** Relationships among agronomical and physiological parameters in finger millet cultivar 1. GY: grain yield, TDW: total plant dry weight, HI: harvest index, PH: plant height, StN: stem number, SpN: spike number, StDW: stem dry weight, SpDW: spike dry weight, NDVI1: greenseeker reading 1, NDVI2: greenseeker reading 2, N: total nitrogen, $\delta^{15}N$: stable nitrogen isotope composition, C: total carbon, $\delta^{13}C$: stable carbon isotope composition, Chl: Chlorophyll, Flav: flavonoid, Anth: anthocyanin, and NBI: Nitrogen Balance Index. Pearson correlation coefficient (r).

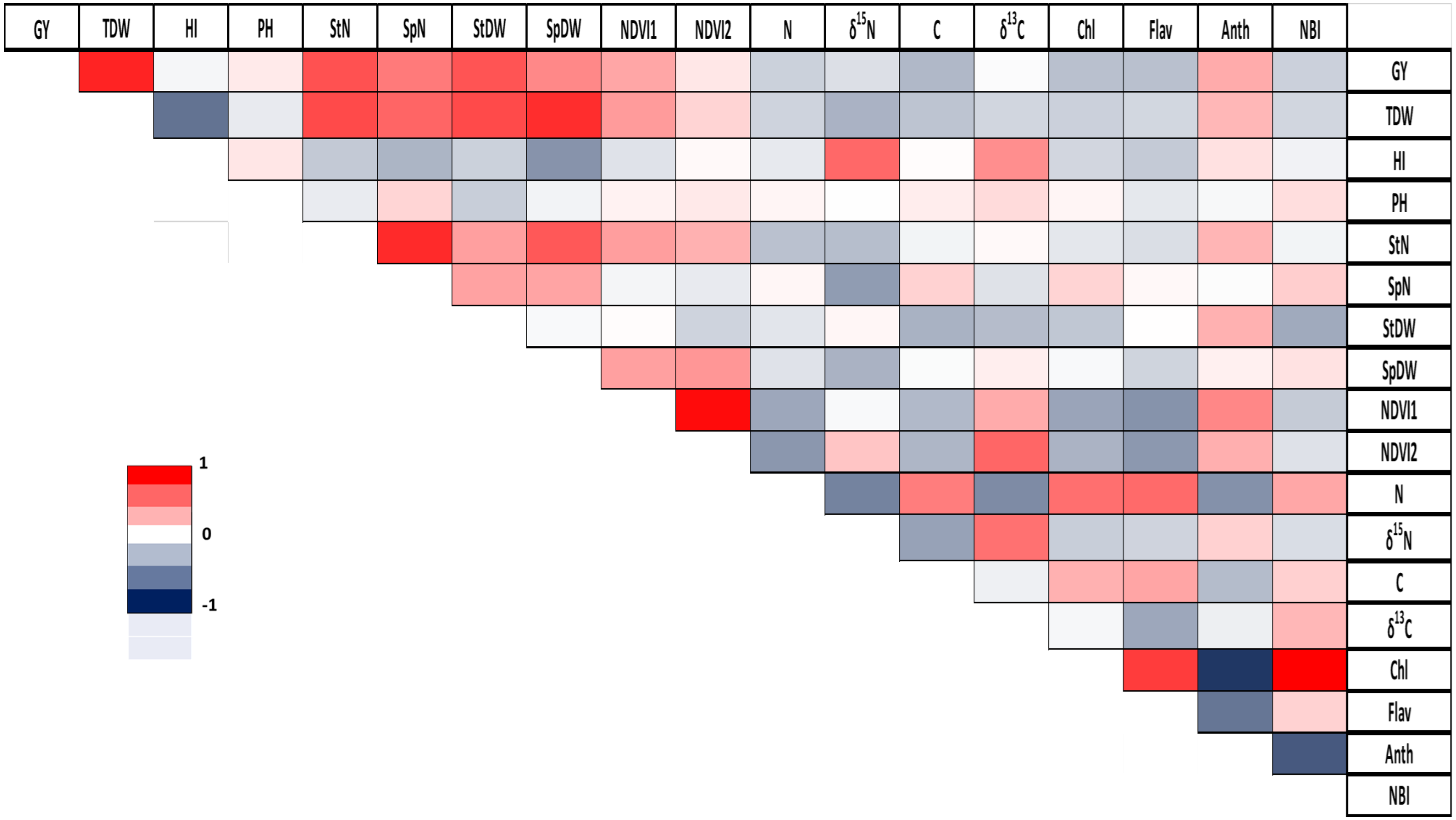

**Figure S9.** Relationships among agronomical and physiological parameters in finger millet cultivar 2. GY: grain yield, TDW: total plant dry weight, HI: harvest index, PH: plant height, StN: stem number, SpN: spike number, StDW: stem dry weight, SpDW: spike dry weight, NDVI1: greenseeker reading 1, NDVI2: greenseeker reading 2, N: total nitrogen, $\delta^{15}N$: stable nitrogen isotope composition, C: total carbon, $\delta^{13}C$: stable carbon isotope composition, Chl: Chlorophyll, Flav: flavonoid, Anth: anthocyanin, and NBI: Nitrogen Balance Index. Pearson correlation coefficient (r).

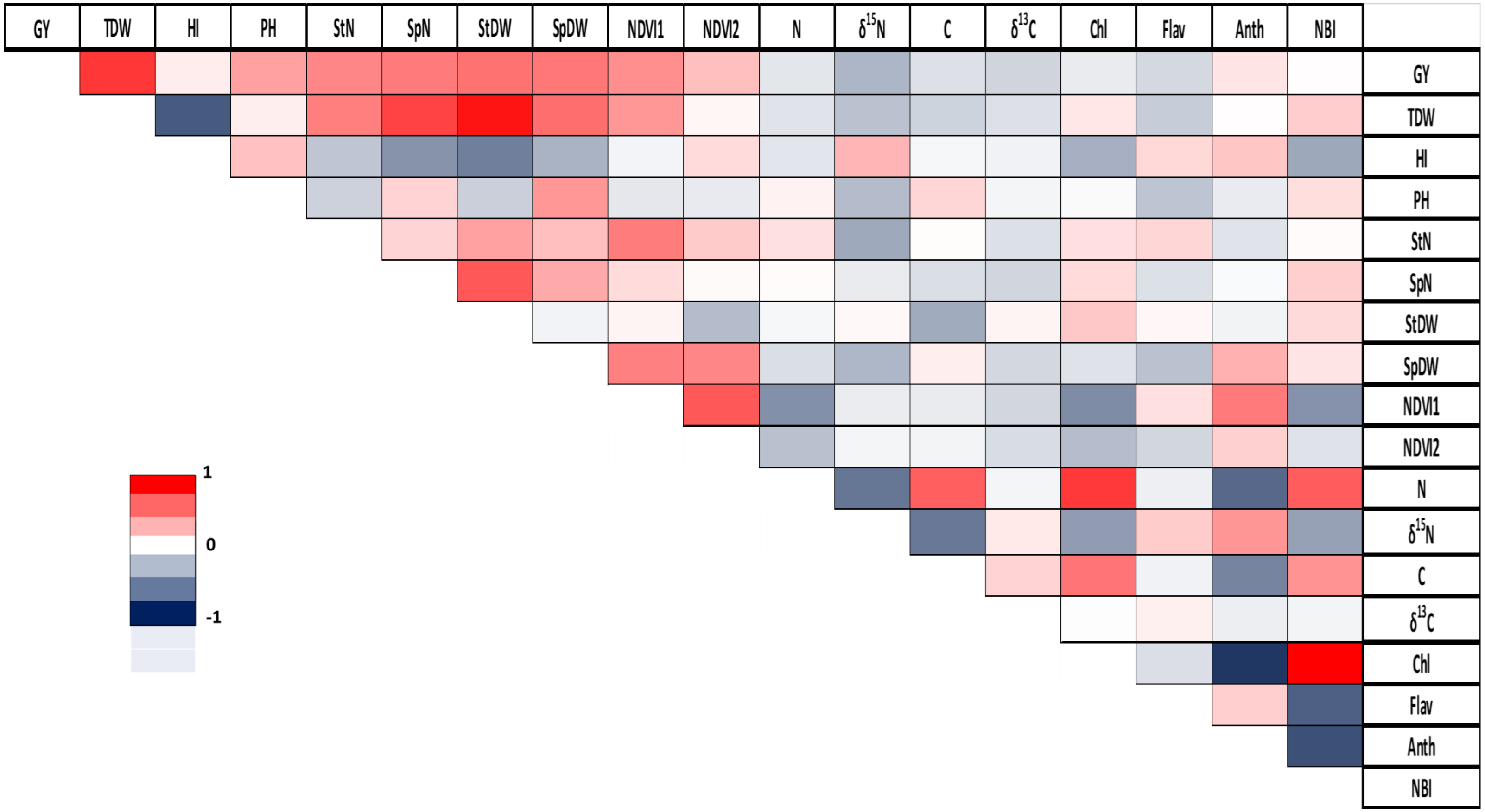

Supplement: Supplementary file 1 [file DataSheet1.pdf]
